# Molecular mechanisms of tubulogenesis revealed in the sea star hydro-vascular organ

**DOI:** 10.1038/s41467-023-37947-2

**Published:** 2023-05-09

**Authors:** Margherita Perillo, S. Zachary Swartz, Cosmo Pieplow, Gary M. Wessel

**Affiliations:** 1 Department of Molecular, Cellular Biology and Biochemistry, BioMed Division, Brown University, 185 Meeting Street, Providence, RI 02912 USA; 2 Whitehead Institute for Biomedical Research, 455 Main Street, Cambridge, MA 02142 USA; 3 Present Address: Marine Biological Laboratory, 7 MBL Street, Woods Hole, MA 02543 USA

**Keywords:** Embryogenesis, Developmental biology, Evolutionary developmental biology, Organogenesis

## Abstract

A fundamental goal in the organogenesis field is to understand how cells organize into tubular shapes. Toward this aim, we have established the hydro-vascular organ in the sea star *Patiria miniata* as a model for tubulogenesis. In this animal, bilateral tubes grow out from the tip of the developing gut, and precisely extend to specific sites in the larva. This growth involves cell migration coupled with mitosis in distinct zones. Cell proliferation requires FGF signaling, whereas the three-dimensional orientation of the organ depends on Wnt signaling. Specification and maintenance of tube cell fate requires Delta/Notch signaling. Moreover, we identify target genes of the FGF pathway that contribute to tube morphology, revealing molecular mechanisms for tube outgrowth. Finally, we report that FGF activates the Six1/2 transcription factor, which serves as an evolutionarily ancient regulator of branching morphogenesis. This study uncovers distinct mechanisms of tubulogenesis in vivo and we propose that cellular dynamics in the sea star hydro-vascular organ represents a key comparison for understanding the evolution of vertebrate organs.

## Abstract (web summary)

Understanding how cells organize into organs is a key effort in developmental biology. Here the authors introduce the sea star hydrovascular organ as a genetically tractable system to understand the contribution of cell migration and signaling pathways in tubulogenesis.

## Introduction

The coordinated organization of cells into a precise three-dimensional architecture is critical for establishing functional organs in development. Tubulogenesis, the process by which hollow tubes form, is an important transient step in the development of many organs, including the neural tube, heart and pancreas^1^. In addition, many vital organs are ultimately organized into tubular shapes with the essential scope of transporting fluids, gasses, or cells, as in the case of kidneys, mammary glands, and vasculature^2^.

A great diversity of mechanisms are used to form hollow tubes in animals, including how cells reach their final position and distinct external cues that guide the proper organ shape^1,2^. Abnormalities in these processes can cause congenital disorders, dysfunctional or displaced organs, and loss of organ symmetry. Yet, because of the great diversity of tubular structures, the general and conserved mechanisms of tube formation in organogenesis are not completely understood^1,2^.

Our knowledge of tube formation is largely derived from specific organs within selected model systems, including *D. melanogaster*, *C. elegans*, *C. intestinalis*, zebrafish, mice, cell culture, and organoids^3–7^. However, there are some key differences amongst these systems. For example, while it is broadly true that cells within tubular epithelia must become polarized, an essential step for lumen formation, polarization is acquired differently in diverse organs and species^4^. Similarly, cell migration, morphogenetic movements, and mitotic division are important for forming the three-dimensional structures. However, each of these parameters contribute to different extents in different species. For instance, while in *Drosophila* tubular organs cells undergo massive proliferation before migration, in vertebrates cell proliferation and migration are coupled^6,8–10^. From an evolutionary perspective, it is therefore an important challenge to determine whether these different cell mechanics in organogenesis reflect the evolution of organs, and whether a basal tubulogenesis toolkit may exist.

Similarly, it remains unclear whether there exists a core and conserved network of signaling pathways that guide cell movements and fate decisions in tubulogenesis. For example, in angiogenesis and vasculogenesis, Notch signaling regulates the differentiation of vascular endothelial cells^11^. However, whether Notch participates in tube formation in other contexts remains poorly understood. In addition, Wnt signaling controls the development of branching organs with complex architectures in both vertebrates and invertebrates^12,13^. However, because of the complexity of these organs, other morphological aspects besides branching have been difficult to investigate. Furthermore, FGF signaling is important for tubulogenesis across species, including the mouse lungs and kidney^14,15^. Nevertheless, many critical aspects of FGF action are still poorly understood, including the downstream targets of FGF signaling, and the conservation of these target genes across organ types and species.

**Fig. 1:**
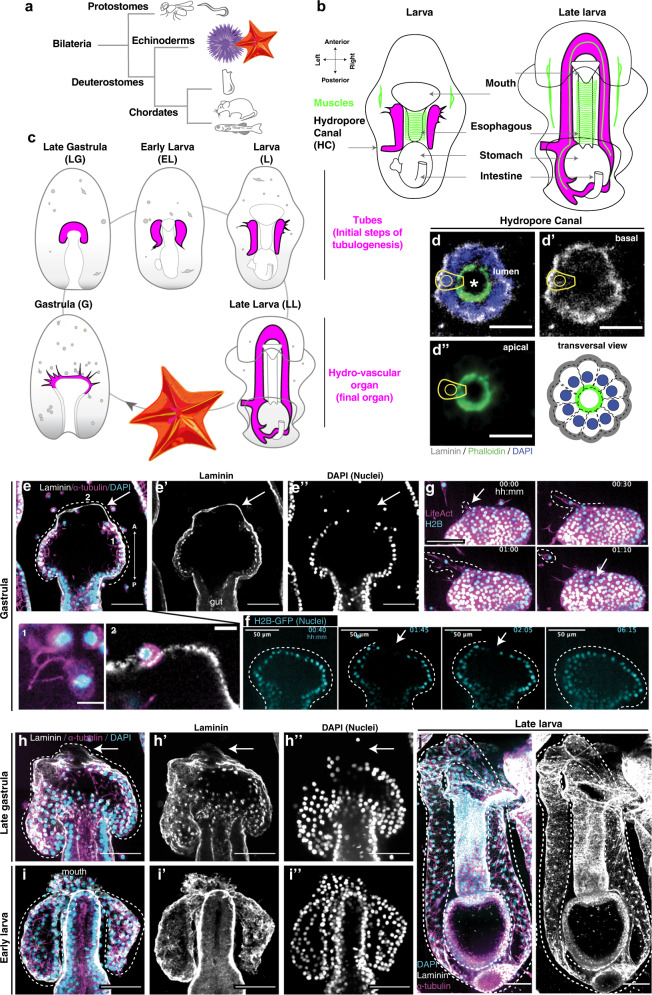
Development of the sea star larva hydro-vascular organ. **a** Phylogenetic relationships of the main bilaterian groups. **b** Summary of sea star larval development with a focus on the hydro-vascular organ (in magenta). The hydro-vascular organ comes from mesodermal precursors located at the tip of the growing gut in gastrula (G). Tubulogenesis starts in the late gastrula (LG), continues in the early larva (EL) and larval stages (L1 to L3). In larval stages, the initial tubes that form the organ elongate posteriorly. The left tube forms the hydropore canal, an opening toward the outside environment. The organ is fully grown in late larva (LL), when the left and right tubes merge to form the closed system of the hydro-vascular organ. **c** Summary of the main larval tissues and organs. **d**–**d”** Transversal view of the tube trough the hydropore canal showing that the epithelium is polarized with actin on the apical side of the cells (facing the lumen) and laminin on the cell basal side. Dotted lines indicate the polarity of a single tube cell. Scale bars 10 μm. **e**–**e’** Laminin staining showing the basal lamina protrusion (arrow) in gastrulae. Alpha tubulin marks cilia in the tube lumen (Insert 1); a cell in between the basal lamina (insert 2, a different z-stack of Fig. 1 E). **f** Single stacks from a time lapse showing regions of the anterior-end of the growing tubes lacking cells (arrows). **g** Single frames from Supplementary Movie 3 showing a cell extending actin-rich protrusions and leaving the epithelium. **h**–**h”** Laminin protrusions still present at late gastrula (arrows). **i**, **j** At larval stages tubes are completely separated from the gut and the laminin layer is continuous. All experiments were independently repeated at least 3 times with similar results. d**:** scale bar 10 μm; **e**, **g**–**j**: scale bars 50 μm. A anterior, P posterior.

The canonical model systems from which most of our knowledge of tubulogenesis derives generally belong to two main phylogenetic groups: ecdysozoans (e.g. worms and flies) and chordates (e.g. tunicates and vertebrates) (Fig. 1a). Echinoderms, which include sea urchins and sea stars, are part of the sister group to chordates and as such occupy a critical phylogenetic position to understand evolution of vertebrate organs, while offering a series of experimental advantages. Here, we take advantage of the sea star *Patiria miniata* as a powerful system to study tubulogenesis within an intact organism. The sea star larva is genetically tractable, optically transparent, and forms a tubular coelom organ called the anterior coeloms, coelomic sacs, or the hydro-axocoel (hydro-vascular organ from here on)^16^. The hydro-vascular organ functions as a hydrostatic skeleton to allow the larva to balance in the water column, and as such it is a simple, but vital organ^17^.

In this work, using live imaging and functional approaches, we identify the intrinsic (cell migration and proliferation) and extrinsic (external cues) factors driving the first steps of tubulogenesis in this system. We reveal that in an early branching deuterostome, directional cell migration coupled with cell division drives tubulogenesis. The initial tube outgrowth, the oriented elongation, and the differentiation of mesodermal cells as epithelial tube cells rely of the FGF, Wnt and Delta/Notch pathways. Our work defines a basic toolkit from where the chordate tubular organs might have evolved.

## Results

### The hydro-vascular epithelium polarizes in early development

Tubulogenesis is a highly dynamic process of individualized cell movements and tissue-level morphogenesis. To define the nature of sea star tube formation, we took advantage of the optical clarity of the larvae and developed techniques to immobilize and live image morphogenesis continuously from gastrulation to early larval stages (Supplementary Movie 1). To standardize the different steps of tubulogenesis, we defined a naming scheme to summarize the critical stages of tube development (Fig. 1b, Supplementary Fig.1). The hydro-vascular organ derives from mesodermal progenitors^18^ that, from the tip of the growing gut, divide into two bilateral tubes which grow towards the posterior end of the larva (Supplementary Movie 2). The two tubes ultimately merge to form a contiguous system with one opening towards the external environment, the hydropore canal (Fig. 1c, d–d”). This entire process happens in ~15 h, as shown in Supplementary Movie 1.

Our first goal was to define the tissue morphologically. A fundamental feature of tubular organs is the presence of a polarized epithelium, so we tested whether the cells displayed apical-basal polarity by visualizing actin and the basal lamina component laminin. A transverse view through the hydropore canal showed that the tube epithelium is polarized, with the apical side of the cells rich in actin cytoskeleton facing the lumen and a basal lamina delimiting the blastocoel environment (Fig. 1d–d”). We found that the tubes were continuously enwrapped by a layer of laminin from where they originated (Fig. 1e–j). At the onset of tubulogenesis (gastrula stage), before the two tubes fully separated from the gut, cells were already polarized, as shown by the presence of cilia that defines the apical surface on the luminal side (Fig. 1e insert 1). Although the basal lamina was generally contiguous with the tube epithelium, we observed a laminin protrusion on the anterior side of the tube that was devoid of cells (Fig. 1e–e”, f arrows). This region where cells are temporarily lacking was also observed in live samples (Fig. 1f). A closer examination showed that some cells were within this laminin layer (Fig. 1e insert 2). With live imaging of actin and nuclei, we observed the dynamic movements of epithelial cells on the anterior side of the growing tubes that migrate out of the epithelium towards the blastocoel (Fig. 1g and Supplementary Movie 3), suggesting that cells left the laminin network on the anterior side. Similarly, areas devoid of laminin on the anterior region were observed until the late gastrulae (Fig. 1h–h”). In larvae, the two tubes are fully separated from the gut and the laminin layer fully covered the tubes (Fig. 1i, j). In the fully-formed tubes, we detected cilia beating (Supplementary Movie 4) suggesting that fluids are circulating inside the lumen of the tubes and that these are not primary cilia. These results overall indicate that the progenitors of the hydro-vascular organ are polarized early in the development of this structure and form a dynamic epithelium.

### Tubulogenesis involves directional cell migration

**Fig. 2:**
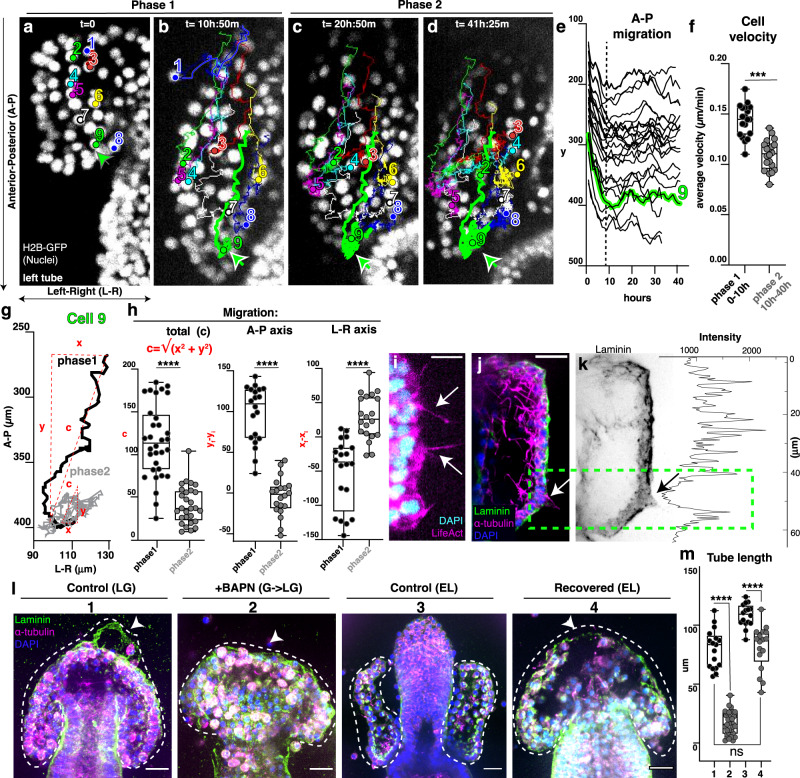
Tube extension and expansion involve active cell migration. **a**–**d** Time lapse images of the tubes expressing H2B-GFP to track movements of single nuclei (Supplementary Movie 6). Tracked cells are labeled with unique colors/numbers to visualize trajectories. In 2D projections, cell movements are recorded along the A-P axis (advancing direction) and the L-R axis. Note that in A and B, cell 1 has undergone an epithelial to mesenchymal transition (EMT) and migrates into the blastocoel, away from the elongating tube. **e** Cell migration along the A-P axis of the embryo (the y axis of the movie) over time. Cell 9 from is highlighted in green to show the two phases of migration. Graph includes cell trajectories of both tubes for 2 independent movies, (Supplementary Movie 5–8; *n* = 30 cells). **f** Average cell velocity for phase 1 and 2 for 2 independent movies. n = 19 (phase 1) and 20 (phase 2); ***p = 0.0008. **g** The trajectory of Cell 9 exemplifies cell movement along A-P and L-R axes. c represents the total cell displacement and is calculated as shown. **h** Maximum cell displacement happens in phase 1 and occurs along the A-P axis. In phase 2, cell displacement happens along the L-R axis. Track data for *n* = 50 cells from two independent movies. **i** Image from Supplementary Movie 9 showing actin-rich filopodia from tube cells (arrows), sb = 10 μm. **j**, **k** Cell protrusion marked by tubulin extend out from a gap in the basal lamina (arrows and green dotted box); sb = 10 μm. Graph indicates intensity of laminin antibody staining to highlight that where the cell protrusion extends out there is a drop in laminin signal intensity. **l** Pharmacological approach to block ECM remodeling inhibits tube extension; sb = 20 μm. **m** Tube length quantification in BAPN treatments. *n* = number of larvae is 17 (1); 34 (2); 16 (3); 17 (4). For all graphs, statistical significance was assessed by a two- sided Student’s *t*-test (****p < 0.0001; ns not significant). In box plots the median is the middle line, box represents 25th and 75th percentiles, whiskers indicate the minimum and maximum data range. Data are presented as mean values +/− SEM. Source data are provided as a Source Data file.

By live imaging, we determined that the tubes elongate from cells at the top of the growing gut (Supplementary Movie 1). We tracked single-cell movements from late gastrula to early larva stage in embryos expressing H2B-GFP to label nuclei. We recorded cell movements with respect to the anterior-posterior axis (A-P) and the left-right axis (L-R) (Supplementary Movie 5–8 and Supplementary Fig. 2a, b). With this approach, we identified two distinct phases of cell migration. In the first phase, cells made substantial movements towards the posterior end of the embryo (Fig. 2a, b, e, g). On average, cells made the most progress along the A-P axis and completed most of their overall movements during this first phase, calculated as the total migration (Fig. 2h). In the second phase, cells maintained their relative positions along the A-P axis (Fig. 2c, d, e–g) and moved on the L-R axis more than on the A-P axis (Fig. 2h). We thereby uncovered a two-phase program of cell movements, in which the cells first move towards the posterior axis to promote rapid tube elongation, and then along the L-R axis to promote tube expansion.

Active cell migration is associated with protrusions rich in actin, including lamellipodia and filopodia^19^. To further assess active cell migration we visualized actin-rich protrusions over several hours with LifeAct and we observed the presence of dynamic filopodia projecting from the tube epithelial cells during migration (Supplementary Movie 9 and Fig. 2i). These filopodia extend from the cell edge rich in actin that also changes dynamically, indicating active cell migration. Tubes are surrounded by a layer of laminin (Fig. 1h) that links epithelial cells to collagen in the extracellular matrix^20^. This layer is not uniform and there are multiple areas where laminin is missing, and where cell protrusions expand out (Fig. 2j, k). To test whether a plastic basal lamina is necessary for tubulogenesis, we inhibited collagen cross-linking with beta-aminopropionitrile (BAPN), an inhibitor of the enzyme lysyl oxidase. In embryos where ECM remodeling was blocked, the laminin protrusion that normally forms anteriorly was missing and tubulogenesis did not begin (Fig. 2l 1 and 2, m). However, when the drug was removed the laminin protrusion reappeared and tubes slowly started to grow back (Fig. 2l 3 and 4, m). Altogether these results suggest that the two phases of tube extension and expansion involve active cell migration that ECM remodeling is also required for tubulogenesis.

### Mitotic growth zones contribute to tube elongation

**Fig. 3:**
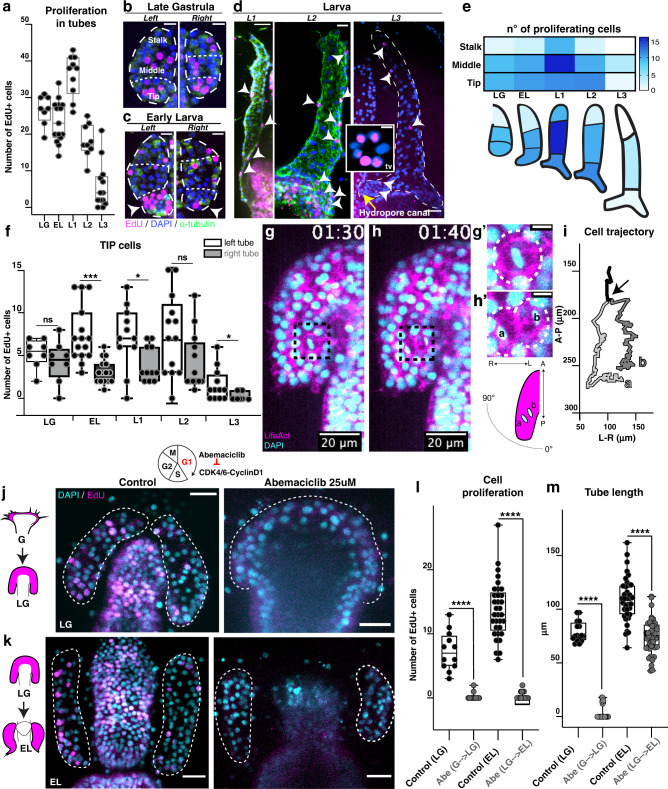
Cell division during tube outgrowth. **a** EdU pulse experiments measure frequency of cell proliferation during tubulogenesis from tube outgrowth to larva stages (as defined in Supplementary Fig.1). Number of total larvae analyzed *n* = 60. **b**–**d** EdU staining (magenta) in the three tube areas: stalk, middle and tip cells. Arrowheads in (**c**) and (**d**) indicate nuclei with incorporated EdU. Insert in (**d**) shows a transverse view (tv) of the hydropore canal from another specimen with nuclei stained with EdU. Scale bars 10 μm (**b**, **c**), 20 μm (**d**), 5 μm (insert). **e** Heatmap showing frequency of cell proliferation (white is zero, dark blue is max). **f** Frequency of cell proliferation in the tip cells for left and right tubes. *n* = 60 total number of larvae. Two-sided Student’s *t*-test: EL (***p-value = 0.0002), L1 (*p-value = 0.0260), L3 (*p-value = 0.0409). **g**, **h** Examples of a dividing cells from Supplementary Movie 10; magnification of a mitotic cell (**g’**) and the same cell undergoing cytokinesis (**h’**); sb = 5 μm. **i** Trajectory of a cell that divides, taken from Supplementary Movie 10. Arrow indicates the mitotic event, where a and b are the two daughter cells. **j**, **k** EdU staining in larvae treated with Abemaciclib to arrest cells in G1 from gastrula to late gastrula (during tube outgrowth) or from late gastrula to early larva (during tube elongation). Dotted lines indicate the tubes. Scale bar 20 μm. **l** EdU incorporation in presence of Abemaciclib to test inhibition of cell cycle and (**m**) effects on tube outgrowth and elongation. Number of larvae *n* = 16 (control LG); 32 (Abe G- > LG); 36 (control EL); 43 (Abe LG- > EL). LG (late gastrula); EL (early larva); L1 (larva stage 1); L2 (larva stage 2); L3 (larva stage 3). Statistical significance was assessed by a two- sided Student’s *t*-test (****p < 0.0001; ns not significant). In box plots the median is the middle line, box represents 25th and 75th percentiles, whiskers indicate the minimum and maximum data range. Source data are provided as a Source Data file.

We next tested whether mitosis contributes to construction of the hydro-vascular organ by quantifying the relative frequency of mitosis along the tubes. Using EdU pulse experiments, we found that the overall cell proliferation along the tube increased from LG until L1 (first larval stage) and then sharply decreased until the L3 (last larval stage) (Fig. 3a). We next asked whether cell proliferation occurs stochastically along the tube, or in spatially localized growth zones. We therefore quantified EdU positive cells within three regions of the tube: the stalk (anterior, where cell migration starts, close to the growing gut), the middle, and tip cells (posterior-most) (Fig. 3b, d and Supplementary Fig. 3a–c). In the initial stages of growth, late gastrula and early larva, cell proliferation was higher in the middle and tip cells than the stalk cells (Fig. e). In the first larva stages (L1-L2), the middle and tip zone cells were dividing the most, whereas by L3 stage, proliferation had decreased throughout the tube (Fig. 3d, e).

The left and right tubes are morphologically distinct, with the left tube specifically branching out to create the hydropore canal, and so we tested whether there were proliferative differences between them. We found that the tip cells of the left tube proliferated more than the right one, from the EL to L3 stages (Fig. 3f). Cell proliferation was particularly active in the hydropore canal (Insert in Fig. 3d, transversal view). We found no difference in cell proliferation at the middle zone between left and right tubes (Supplementary Fig. 3E). In the late larva, cell proliferation occurred exclusively in the posterior-most region of the final tube organ (Supplementary Fig. 3f). In summary, an extensive cell division during tubulogenesis occurs within specific zones of the middle and tip areas of the tubes, and especially within the left tube tip.

We next asked whether oriented cell division could underly the directional elongation of the tubes. We first measured the angle that mitotic cells make respect to the direction of growth (the anterior-posterior axis). If orientated cell division was a driving force for tube growth, we predicted a division angle close to 0° during phase 1 (A-P growth, tube elongation) and close to 90° in phase 2 (L-R growth, tube expansion). We found that the orientation of cell division is non-random, occurring with an average angle of 36.9° degrees with respect to the A-P axis (Fig. 3g–h’, Supplementary Fig. 3g), with no significant difference between phase 1 and phase 2 (Supplementary Fig. 3h). Despite the orientation of the division plane, after mitosis completes, the daughter cells do not follow the direction of the angle they divided but instead continue migrating first along the A-P axis and then along the L-R axis (example in Supplementary Movie 10). These results support the occurrence of oriented cell division, but it does not correlate with the direction of tube growth. We therefore suggest that cell movement in the tubes is distinct from cell division, and rather represents an active migration process. The observed oriented cell division may instead function in another aspect of organ formation, such as expansion of the tube diameter.

We next asked whether cell division was required for tubulogenesis by blocking cell cycle progression in G1 using the Cdk4/6 inhibitor Abemaciclib (LY2835219)^21^. First, to test whether cell proliferation was necessary for initial tube outgrowth, we added the inhibitor from the end of gastrulation until the early larva stage. Importantly, and consistent with a G1 arrest and failure to replicate DNA, the tube cells did not incorporate EdU under these conditions (Fig. 3j–l). When cell cycle progression was blocked, tube outgrowth was fully prevented (Fig. 3j, m). Second, to test whether ongoing cell division was needed after initial outgrowth to support continued elongation, we instead treated embryos from late gastrula to early larva (Fig. 3k). We found that cell cycle arrest at this stage prevented further elongation, with the length of the tubes remaining the same as when the drug was added (Fig. 3m). These results indicate that the cells that make the organ are not completely segregated early in embryogenesis but rather are generated by continual cell proliferation. Thus, the number of cells in the tube epithelium increases over time and this cell proliferation is critical to both initiate and sustain tubulogenesis.

### Delta-Notch signaling directs cell-fate decisions

**Fig. 4:**
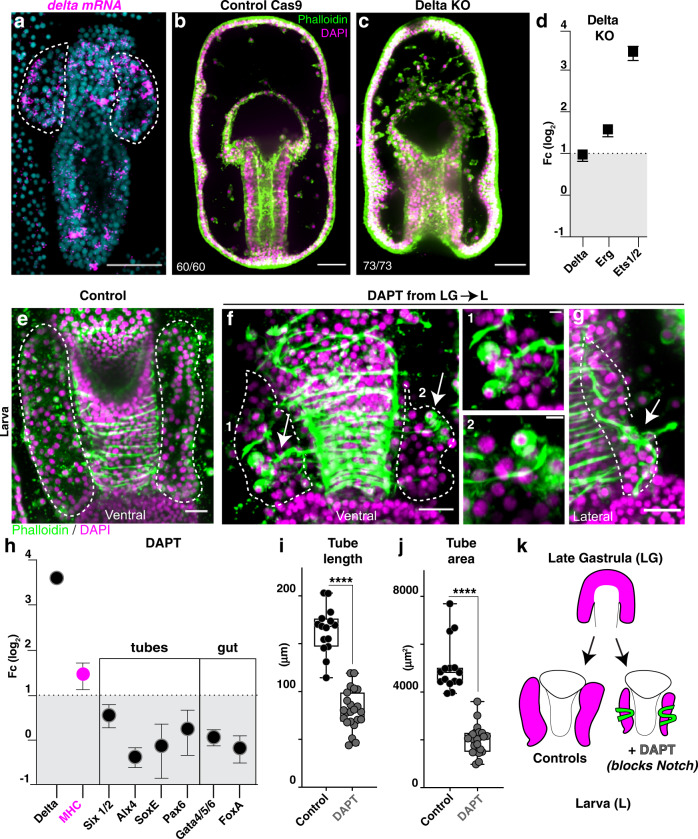
Delta-Notch signaling represses a muscle phenotype in the tubes. **a** Fluorescent in situ hybridization (FISH) shows Delta gene expression in scattered cells of the gut and the tubes (highlighted by dotted lines). **b**, **c** Delta knock out embryos fail to form the epithelial tubes that instead become excess mesenchymal cells. Penetrance of the Delta KO phenotype was 100% and all embryos died at the late gastrula stage; for genomic mutation see Supplementary Fig.12. **d** qPCR showing increase of mesodermal markers *Erg*, *Ets1/2* and *Pax6* in delta KO embryos. Bars represent mean with standard error of the mean (SEM)**. e**–**g** Larvae stained with phalloidin to mark muscles; Delta-Notch inhibition leads to smaller tubes constricted by muscle fibers that wrap around the tubes (arrows indicate muscles, magnification in inserts 1 and 2) and the foreguts. Arrows in g show that tube cells become muscle-like. Note that the control tubes are as long as the esophagus, while in treated embryos the tubes are shorter than the esophagus**. h** The increase in muscle-like cells is also reflected by increase in expression of the muscle marker MHC, while other known marker genes for tubes and the digestive system do not change. Bars represent mean with standard error of the mean (SEM). *n* = 3 biologically independent experiments. **i**, **j** Tubes are significantly smaller in DAPT treated than control embryos. Control: *n* = 15 larvae; DAPT treated *n* = 23 larvae. **k** Cartoon summarizing the result of Notch inhibition. All experiments are representative of 3 biological replicates. **a**, **b**, **c**, **f**, **g** scale bar = 50 μm. **h** scale bar is 10 μm. Statistical significance was assessed by a two- sided Student’s *t*-test (****p < 0.0001). In box plots the median is the middle line, box represents 25th and 75th percentiles, whiskers indicate the minimum and maximum data range. Source data are provided as a Source Data file.

Having defined that epithelial cells migrate, and that mitosis is an essential intrinsic force for tube development, we next tested the contribution of extrinsic signaling pathways. In the sea star embryo, Delta-Notch signaling is critical during gastrulation for specifying all mesodermal cell types, including the hydro-vascular organ precursor cells, migratory mesoderm, and muscles^22,23^. To test whether Delta-Notch signaling could also guide later steps of tube formation we first examined the spatial expression of the Delta ligand after gastrulation and detected Delta mRNA in different tissues, including most tube cells (Fig. 4a). The Notch receptor was instead expressed ubiquitously (Supplementary Fig. 4a), as also reported in other studies^22^. To define the contribution of the Delta+ tube cells to tubulogenesis we generated Delta knockout embryos. In Delta KO late gastrulae, the tube was not specified and there was an increase of mesodermal cells expressing *Erg* and *Ets1/2* (Fig. 4b–d), markers of mesenchyme cells^22,23^. These results are consistent with previously reported pharmacological Notch inhibitions^22^.

To test the contribution of Delta+ cells to later stages of tubulogenesis, we used the γ-secretase inhibitor DAPT to block Delta/Notch signaling with temporal control^22,24^. When we treated embryos at the end of gastrulation with DAPT, after tube cells were already specified, Delta-Notch inhibited larvae displayed increased muscle fibers wrapped around the tubes, even resulting in increased constriction (Fig. 4e–g, Supplementary Fig. 4b). These muscle fibers appeared to originate from the tube itself (arrows in Fig. 4f, g, inserts 1 and 2). In further support of excess muscle specification in the absence of Delta/Notch signaling, we detected increased expression of the muscle marker myosin heavy chain (*MHC*) (Fig. 4h). In addition, the treated larvae had short and thin tubes (Fig. 4i, j). These results suggest that continued Delta/Notch signaling is important to restrict excess muscle specification, and perturbations of this pathway results in thinner tubes.

We next asked how gene expression in the tubes was affected under Delta/Notch inhibition. We tested a panel of known genes expressed in the tubes in this animal, including *Six1/2*, *SoxE*, *Alx4* (*Alx1l*), and *Pax6*^18,22,25^. We found that the expression of these genes was unchanged, despite the tubes being smaller (Fig. 4h). Some Delta+ cells in the digestive system expressed the genes *FoxA* and *GataE*^23^, which we also found to be unaffected. As seen in previous studies^22,23^, Delta mRNA expression was increased when Delta/Notch signaling is blocked, indicating that the perturbation was effective (Fig. 4e). These data suggest that although early Delta/Notch signaling is required for tube cell specification^22,23^, known markers of the tubes were not affected by later stage Notch inhibition. Instead, we found that Notch signaling at later stages prevents the uncontrolled specification of muscle-like cells from the tube cells (Fig. 4k).

### Wnt signaling guides directional growth of the tubes

**Fig. 5:**
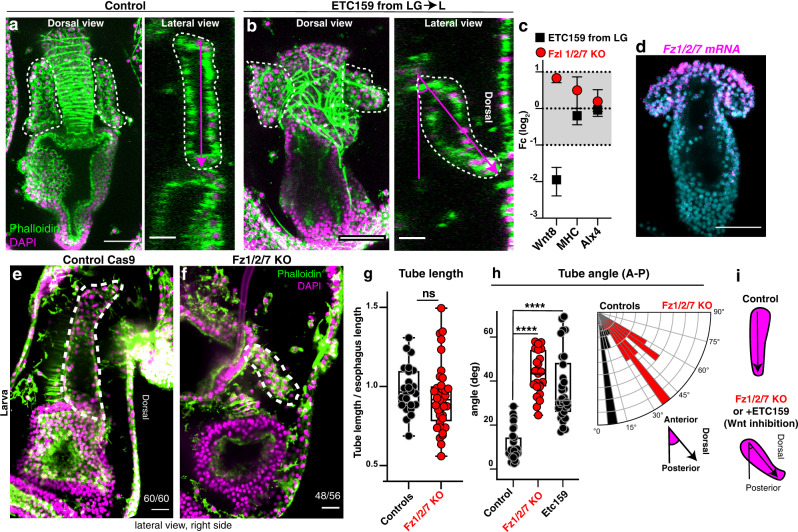
Wnt signaling directs tube orientation through the Fzd1/2/7 receptor. **a**, **b** Wnt inhibition with ETC159 from late gastrula to L2 caused a change in orientation of the tube outgrowth from anterior-posterior to anterior-dorsal. Confocal z-stacks are dorsal views and lateral views obtained from orthogonal projections of the same larvae. **c** qPCR shows Wnt8 gene expression decreases when ETC159 (that block secretion of Wnts) is used. Bars represent mean with standard error of the mean (SEM). n = 3 biologically independent experiments. **d** FISH showing that the Wnt receptor Fzd1/2/7 is expressed in the tube cells in early larvae. **e**, **f** In control larvae tubes grow parallel to the anterior-posterior axis, while in Fzd1/2/7 knock out larvae the tubes grow towards the dorsal side and recapitulate the same tube orientation defects seen with ETC159 treatment. Percentage of the larvae showing the Fz1/2/7 KO phenotype was 86%; for genomic mutation see Supplementary Fig.13. **g** Tube length measured as a ratio of tube length/esophagus length; *n* = 24 larvae for controls, *n* = 36 larvae for Fz1/2/7 KO. **h** Tube orientation measured as the angle that the tube makes with respect to the anterior-posterior axis of the larva. Number of tubes measured is *n* = 30 for controls, *n* = 30 for ETC159 treated and *n* = 23 for Fzd1/2/7 KO. Same data shown as a rose plot to indicate difference in orientation. **i** Schematic summarizes the observed phenotypes. All experiments are representative of 3 biological replicates. **a**, **b**, **d**–**f** scale bar is 50 μm. **a** and **b** lateral views are 20 μm. Statistical significance was assessed by a two-sided Student’s *t*-test (****p < 0.0001; ns = not significant). In box plots the median is the middle line, box represents 25th and 75th percentiles, whiskers indicate the minimum and maximum data range. Source data are provided as a Source Data file.

Since the sea star tubes stereotypically extend along the anterior-posterior axis, we asked what signals guide this polarized growth. The Wnt pathway is a broadly conserved regulator of polarity at the organismal level and branching for many tubular organs^12,13^ and inhibition of Wnt signaling in the early stages of sea star development blocks the formation of endomesodermal structures^26^. To test the later contribution of Wnt signaling to tubulogenesis, we blocked secretion of all Wnt ligands after gastrulation with the porcupine inhibitor ETC-159^27^. Importantly, treatment at this stage does not grossly affect primary body axis or endomesoderm specification, which are established earlier in development. We found that in Wnt-inhibited larvae, the tubes elongated towards the dorsal ectoderm instead of posteriorly, as in controls (Fig. 5a, b). As a control, we tested the expression of a known beta catenin target, Wnt8^26^, and found it to be downregulated with ETC-159 treatment, while the expression of tube and muscle associated genes were unchanged (Fig. 5c). Consistent with the broad expression and role of Wnt genes^26^, several developmental defects were observed, including disorganized muscle patterning and straight gut (Fig. 5b). Our data suggest that Wnt signaling drives directional growth of the tubes towards the posterior end of the larva.

We next sought to define the specific Wnt signaling pathway and its receptor used in tubulogenesis. A prior study identified *Fz1/2/7* as initially localized broadly in the tip of the archenteron, and later selectively in the esophagus and in the tubes that form the hydro-vascular organ^28^. We further defined the localization of *Fz1/2/7* mRNA, and found its expression enriched in the anterior tube cells (Fig. 5d). To test whether tube orientation was dependent on Fz1/2/7, we generated Fz1/2/7 knockout embryos and found that the tubes elongated dorsally instead of posteriorly, which phenocopied Wnt inhibition by EC-159 (Fig. 5e, f). This approach was more spatially targeted, as shown by unaltered levels of Wnt8 (Fig. 5c). Although tubes in Fz1/2/7 KO larvae looked smaller than controls, their length relative to the esophagus (that in controls runs parallel to the tubes) was comparable, indicating that in these smaller larvae, the tubes reach their maximum length proportionally (Fig. 5g).

To assess the directionality of tube elongation we measured the angle that each tube formed with the larval anterior-posterior axis and found that this angle is similar in Fz1/2/7 KO and ETC-159 treated embryos but greater than in controls (Fig. 5h). We conclude that Wnt signaling via the Fz1/2/7 receptor guides oriented growth of the left and right tubes towards the posterior end of the larva, the site where the two tubes will eventually fuse to form the hydro-vascular organ of the late larva.

### FGF initiates elongation by controlling cell proliferation

**Fig. 6:**
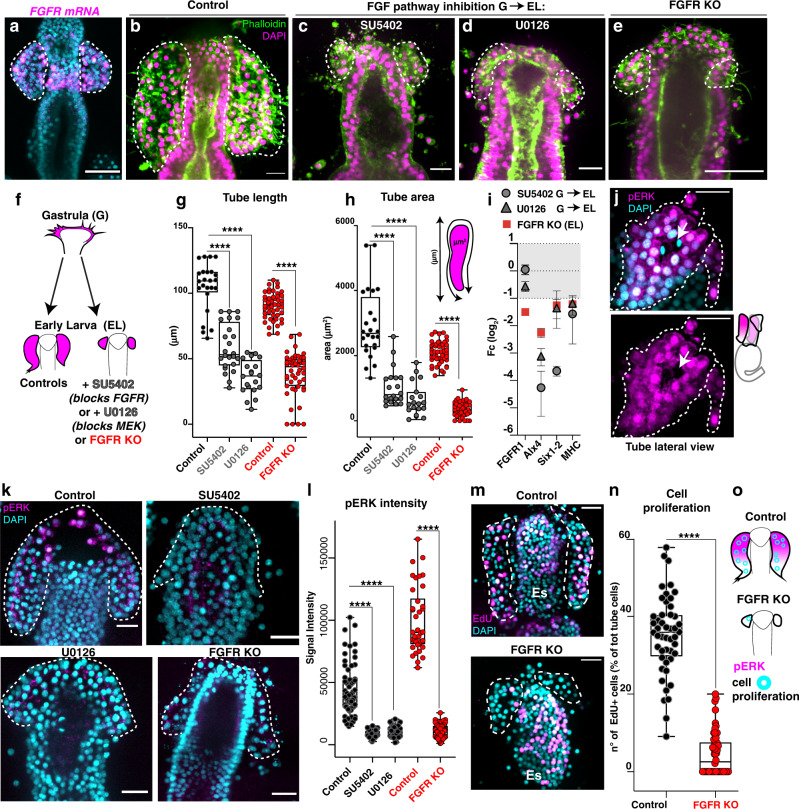
FGF signaling promotes tube outgrowth through pERK. **a** FISH of FGFR shows expression in scattered cells. **b** Control and early larva treated with the FGFR inhibitor SU5402 (30μM) (**c**) or with the MEK inhibitor U0126 (10μm) (**d**) from the end of gastrulation to early larvae (G→EL). **e** Early larvae in which the FGFR is knocked out by Cas9 lack the tubes. Percentage of the FGFR KO larvae showing the phenotype was 96%; for genomic mutation see Supplementary Fig.14. **f** Cartoon summarizing FGFR inhibition phenotypes. **g**, **h** Tube length and area are significantly decreased in larvae where the FGF pathway was blocked. *n* = number of tubes (controls = 26, SU5402 = 26; U0126 = 20, CAS9 control = 48; FGFR KO = 50). **i** qPCR of significant genes in larvae where tube outgrowth was prevented. Bars represent mean with standard error of the mean (SEM). *n* = 3 biologically independent experiments. **j** Immunofluorescence showing that active ERK is localized in the nuclei of most tube cells. Arrows indicate dividing cells without pERK staining. **k** pERK staining is absent when FGF signaling is blocked. **l** Signal intensity of pERK in the above experiments. The *Y* axis in the graph shows the raw integrated intensity for pERK normalized by the background signal for each image. **m** EdU staining to mark proliferating cells and signal quantification (**n**). Since the tubes of the FGFR KO larvae had fewer cells than controls, we assessed proliferation rate by counting the EdU+ cells normalized to the total number of tube cells; n = 48 larvae (controls), n = 50 larvae (FGFR KO). **o** Schematic showing that inhibition of the FGF pathway at different levels prevents tube growth and pERK activation. White dotted lines outline the tubes. Es esophagus. Scale bar = 50 μm (**a**, **e**); scale bar = 20 μm (**b**–**d**, **j**, **k**, **m**). Statistical significance was assessed by a two- sided Student’s *t*-test (****p < 0.0001; ns not significant). In box plots the median is the middle line, box represents 25th and 75th percentiles, whiskers indicate the minimum and maximum data range. Source data are provided as a Source Data file.

FGF signaling has been implicated in the morphogenesis of many kinds of tubular structures^29^. Consistent with a potential role in forming the tubes, we found that the FGF receptor is expressed by scattered tube and esophageal cells (Fig. 6a). To test whether FGF signaling is required for tube formation, we used the FGFR inhibitor SU540^30^. We found that larvae in which FGFR was inhibited following gastrulation had partially missing tubes (Fig. 6b, c). As a second approach, we used the inhibitor U0126 to block phosphorylation of the FGFR downstream effector kinase MEK^31^. This treatment also resulted in a partial lack of tubes, similar to the upstream FGFR inhibition (Fig. 6d). We then knocked out FGFR and found that also with this third approach the tubes developed only partially (Fig. 6e, cartoon in f), with a significant decrease in tube length and area compared to control early larvae (Fig. 6g–h). Moreover, when FGFR was knocked out or if its kinase domain was blocked, genes normally expressed in the tubes and the muscle marker *MHC* were significantly downregulated (Fig. 6i). Aside from lacking the tubes, the overall anatomy of the late larva was normal (Supplementary Fig. 5), indicating a highly selective role of FGF signaling in tubulogenesis. Consistent with inactivation of the FGF pathway, we found that all of these manipulations resulted in a decrease in nuclear pERK, the target of MEK (Fig. 6j, k). After two weeks, while controls were alive, the FGFR KO larvae died, suggesting that lack of the tube organ is critical for larval survival.

Knowing that FGF signaling promotes cell proliferation through pERK^31^, we tested whether FGFR influenced the cell proliferation we observed in the initial steps of tubulogenesis. We performed EdU pulse labeling to mark cells that have synthesized DNA in early FGFR knockout larvae and found a significant reduction of proliferating tube cells (Fig. 6m, n). This reduction in FGF-dependent cell proliferation was specific for the tube cells since cells of the esophagus that also expressed FGFR (Fig. 6a) did not show pERK activity (Fig. 6j, k) and proliferation was not reduced there when FGFR is knocked out (Fig. 6m). We conclude that the FGF signaling pathway initiates tube outgrowth by promoting tube cell proliferation through pERK (Fig. 6o).

### RNA-seq reveals FGF-induced genes during tubulogenesis

**Fig. 7:**
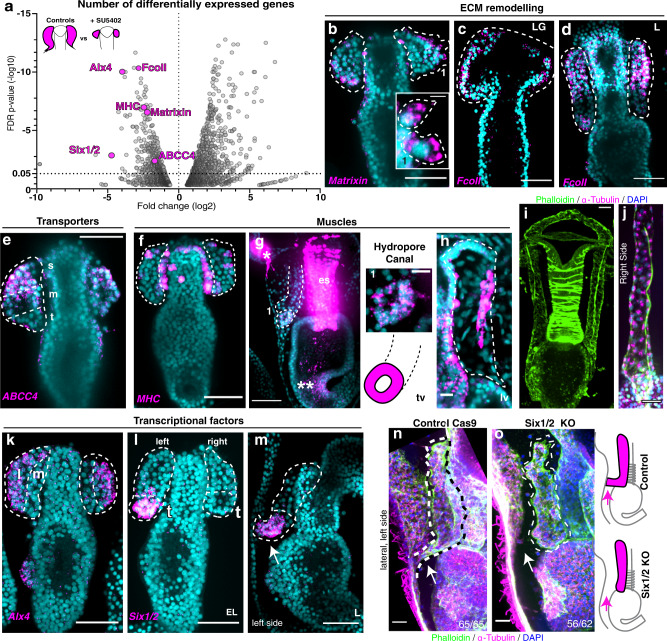
Differential RNA-seq reveals genes responsive to FGF signaling. **a** Volcano plot of differentially expressed genes between controls and larvae where FGFR was blocked. Using the Wald test, *p*-values, false discovery rate (FDR) and fold changes were generated. Gene/Transcripts with *p* value < 0.05 were called as differentially expressed genes/transcripts for the comparison. **b**, **c** FISH for genes involved in extracellular matrix (ECM) remodeling, *matrixin* (**b**), and *Fcoll* (**c**, **d**). Insert 1 in (**b**) shows *matrixin* transcripts in cells extending protrusions. **e** FISH for the transporter *ABCC4* shows expression in tube stalk cells. **f**–**h** FISH showing *myosin heavy chain* (*MHC*) gene expression in the hydropore canal (1), the pyloric sphincter (** in g), the tube longitudinal muscle (h), the dorsal muscles (* in g) and the esophageal muscles (es in **g**). **i**, **j** Longitudinal muscles are localized on the tube ventral side. **k–m** FISH for *Alx4* and *Six1/2*. Six1/2 is expressed at the tip of the left tube and later in the hydropore canal (arrow in m). **n**, **o** Six1/2 KO did not form the hydropore canal (arrow). Percentage of the Six1/2 KO phenotype was 90%; for genomic mutation see Supplementary Fig.15. All images are maximum projections of confocal z-stacks. All experiments were independently repeated at least 3 times with similar results. FDR false discovery rate, s stalk, m medial, t tip, tv transverse view, lv lateral view. scale bar = 50 μm (**b**–**g**, **k**–**o**); scale bar = 20 μm (**i**, **j**); scale bar = 10 μm (**h**). Source data are provided as a Source Data file.

The FGF pathway is involved in multiple aspects of tubulogenesis, but the genes that link it to morphogenesis are still poorly understood^29^. Blocking FGF signaling either genetically or pharmacologically precisely ablates the tube structures in sea star larvae (Fig. 6c–e). To define the landscape of genes activated by the FGF pathway, and potential genes that define cell types of the hydro-vascular organ, we generated tube-ablated larvae by SU5402 treatment soon after gastrulation. We then performed differential RNA-seq on these samples. In the analysis we selected candidates with an FDR SU5402/Controls <0.05 and fold change threshold of ±1.6 log_2_ SU5402/Controls, as used in previous studies^32^. With this approach, our analysis identified 147 downregulated and 346 upregulated transcripts (Fig. 7a, Supplementary Data 4). We then validated this dataset by performing fluorescent in situ hybridization to test transcript localization, and we further investigated the function of selected genes.

As the SU5402 treatment inhibits tube formation, we focused on genes that were downregulated to identify candidates were uniquely expressed in the initial tubes. Most of the downregulated genes were involved in extracellular matrix homeostasis (Supplementary Fig. 6, Supplementary Data 4). For example, we identified the extracellular metallo-protease *matrixin* as a target of FGFR (Fig. 7b, Supplementary Fig. 7). This protease is secreted and its transcripts were enriched in lateral tube cells (Fig. 7b). Our RNA-seq study also identified the secreted Kazal type serine protease inhibitor *FcoII* as a FGFR target, which we found to be enriched in the growing tubes (Fig. 7c, d, Supplementary Fig. 8). Protease inhibitors spatially and temporarily control protease activity to tune ECM remodeling. In the first step of tubulogenesis a transient basal lamina protrusion appears on the anterior side of the tubes and then disappears once the two tubes separate (Fig. 1e, f). To test its role in this remodeling, we knocked down FcoII, and found that this protrusion was stabilized. Moreover, the organ did not grow properly since the two bilateral tubes remained connected on the anterior most-end (Supplementary Fig. 9a).

Another group of genes downregulated in FGFR blocked larvae were transporters. The ATP-binding cassette sub-family C member 4 (MRP4 or ABCC4) is a multidrug resistance-associated protein whose expression in the mouse brain is dependent on FGFR1^33^. However, a role for ABCC4 in other morphogenetic mechanisms is unknown. We found that *ABCC4* was a FGF target and was expressed in the initial tube, particularly in the stalk cells (Fig. 7e, Supplementary Fig.10), suggesting that the link with the FGF pathway is conserved across species, even if the transporter is expressed in different tissues.

Another class of genes downregulated when FGFR was blocked were muscle-related genes, including troponin, myophilin, twist, myosin light and heavy chain (Supplementary Data 4). We found that the muscle marker myosin heavy chain (*MHC*) was initially expressed first by a few tube cells, but once the tubes elongated, they formed two distinct populations of muscles (Fig. 7f, g). The first one was a muscle in the hydropore canal (Fig. 7g, insert 1). Here, *MHC* transcripts marked cells that did not have long fibers, similar to the intracellular contraction apparatus seen in the endodermal muscles of the sea star pyloric sphincter (** in Fig. 7g) and in sea urchin larva pyloric and anal sphincters^34^. The second muscle cells formed longitudinal fibers that appeared in L3 stage (Fig. 7h) and later extended along the whole length of the hydro-vascular organ (Fig. 7i, j). We further investigated the function of the longitudinal muscles we identified with live imaging and found that the twitching of these muscles allowed for the contraction of the hydro-vascular organ (Supplementary Movie 11).

The transcription factors most affected by FGFR inhibition were *Alx4*, that was enriched in the tube lateral cells (Fig. 7k) and *Six1/2*, that was first enriched in the tip of the left tube during tube elongation and later it marked the hydropore canal (Fig. 7l, m, arrow). Because we found that *Six1/2* is expressed in a defined structure of the tube (the opening towards the outside environment), we focused on this gene and found that Six1/2 knockout larvae, while otherwise morphologically normal, did not form the hydropore canal, even 3 days after this structure normally forms in controls (Fig. 7n, o and knock down in Supplementary Fig. 9b). Since the hydropore canal forms by tissue bending at a defined branch point, we propose that Six1/2 activation downstream of FGF signaling is critical for branching morphogenesis. In summary, blocking the FGFR pharmacologically we found a variety of candidate genes that are activated by the FGF pathway and that defined subpopulations of tube cells during tubulogenesis.

## Discussion

The tremendous diversity in strategies to form hollow tubes suggests that either these structures are not homologous, or that the toolkits to build such structures are not conserved. Here, we took advantage of the tubular hydro-vascular organ of the sea star larva to unravel the mechanisms of tubulogenesis in an intact, early branching deuterostome. We show that tube formation and elongation require that cells migrate and divide in defined zones. Cell fate depends on Notch signaling, while tube orientation requires Wnt signaling through the receptor Frizzled 1/2/7. In parallel, cell proliferation downstream of FGF signaling is critical for tube outgrowth and extension. Finally, we find that FGF reception induces the expression of genes potentially involved with hydro-vascular organ function, including muscles, ECM remodeling genes and transcription factors. These observations in the sea star, a representative of the sister group to all other deuterostomes including vertebrates, provide fundamental insight into the evolution of tubular organs.

### A transition in tubulogenesis appeared in deuterostomes

**Fig. 8:**
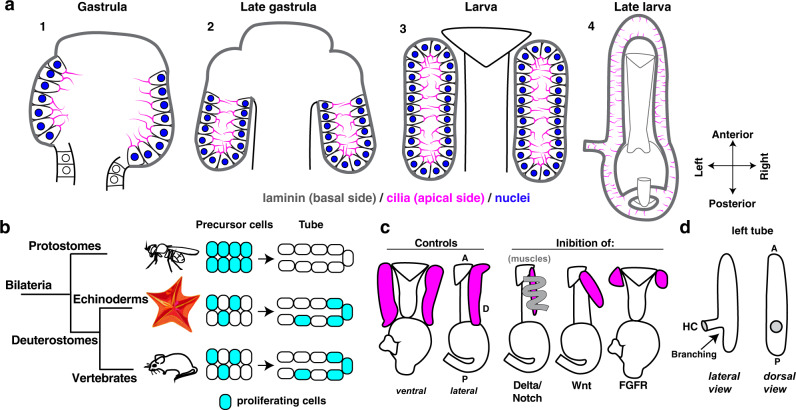
Summary of the intrinsic and extrinsic factors that guide tubulogenesis of the hydro-vascular organ. **a** Stages of sea star hydro-vascular organ development. The precursor cells of the hydro-vascular organ come from mesodermal progenitors at the tip of the growing gut (1). In the first stage of tubulogenesis, pre-polarized cells form a lumen and grow towards the posterior side of the embryo to form two tubes (2). During embryogenesis, left and right tubes detach from the esophagus (3) and finally form a close system where the left and right tube connect anteriorly and posteriorly, the hydro-vascular organ. **b** Phylogenetic tree showing the relationship of the in vivo systems for the study of tubulogenesis. In protostomes tubular organs form by a first step of cell proliferation followed by morphogenesis. In echinoderms and in vertebrates (both deuterostomes) cell proliferation is continuously coupled with cell migration during morphogenesis. **c** Cartoon summarizing the role of signaling pathways on tube formation. **d** Cartoon showing the branch formed by the left tube, the HC (hydropore canal) that opens on the outside environment. A anterior, P posterior, D dorsal.

A fundamental feature of tubular organs is that the constituent epithelial cells are polarized. Tubes can form either from sheets of polarized cells or from nonpolarized cells that can acquire polarization during morphogenesis to form a lumen de novo^10^. We found that the hydro-vascular organ develops from pre-polarized precursor cells, therefore positioning this organ among the first category. Figure 8a summarizes the main steps of the sea star tubular organ development. Mesodermal cells from the tip of the growing gut during gastrulation evaginate to form two initial tubes formed by a simple monolayer epithelium that keeps its polarity during growth. Cells are ciliated and rich in actin at the apical side facing the lumen, while the basal side faces the basal lamina (Fig.1d–g). Similarly, organs like the Drosophila salivary glands and vertebrate lungs also form from polarized precursors that migrate to accomplish tube morphogenesis^2,10^. However, the mechanisms of cell dynamics in tubulogenesis differ between *Drosophila* and mammalian tubular organs. For instance, the *Drosophila* salivary gland and the tracheal precursor cells complete proliferation prior to beginning tubular morphogenesis and only in later stages do tracheal cells divide during tube remodeling^3,8^, while in vertebrate organs like lungs and kidney cell proliferation and migration are always coupled^6,9,10^. However, comparisons among tubular organs are complex since many of these organs have diverse developmental origins, like the fly trachea that is derived from the ectoderm, vertebrate lungs from the endoderm and kidney from the mesoderm^3,7,15^. Moreover, tubular organs can use diverse growth strategies at different stages. Our aim is to define the basic toolkit that gave rose to the deuterostome tubular organs. Are there generalizable differences between vertebrates and invertebrates? Or, does this difference instead reflect the consequence of distinct life histories between individual species?

Here we show that as in vertebrates, cell migration and cell proliferation are coupled during tubulogenesis in the sea star, an early-branched deuterostome (Fig. 8b). To establish the initial tube, cells undergo a two-phase migration from the tip of the growing gut to finally run parallel to the gut itself (Supplementary Movies 5–8). The angle of oriented cell division during tube growth is right in between these two phases of elongation and expansion, an ideal angle to favor the expansion of the diameter of the tube. A question that remains unanswered is whether cell migration is also necessary for tubulogenesis. Cell displacement during tubulogenesis is active, since moving cells have actin-rich filopodia that extend out of the basal lamina that surround the tubes (Supplementary Movie 9). Another hint that cell migration plays a role in tubulogenesis is given by the observation that when collagen crosslinks are blocked, tube outgrowth is prevented (Fig. 2l, m), suggesting that cells migrate along the basal lamina. If cell migration is critical for tube outgrowth, inhibition of the actin-based cell protrusions specifically in the tubes should prevent the tissue from growing. Such experiments will be possible in the future thanks to the new technologies for inducible knock outs^35^ or cell-type specific expression of dominant negative Rho-A, an important actin regulator.

Similarly to tubulogenesis in vertebrates, subsets of cells actively divide during these migrations in the sea star. Mitosis plays a critical role in tubulogenesis, since cell number grows over time and when it is blocked, there is a significant lack of tissue. Localized cell proliferation is an important driver of shape changes, as in many mammalian tubular organs^36^. In the sea star tubes, we find that proliferation is downstream of FGF signaling, and it is concentrated in two main proliferation zones: the middle region of both tubes and the tip of the left tube. We propose that the extensive proliferation in the middle of the tube drives A-P elongation. Instead, proliferation at the tip of the left tube may drive formation of the critical branch point that forms the hydropore canal (Fig. 8c). Indeed, by blocking cell cycle progression by Cdk4/6 inhibition, or FGF perturbations at different time points, tube initiation or elongation was disrupted. FGF signaling regulates proliferation in the mouse Wolffian duct^14^, in the kidney metanephric mesenchyme^37^, and in the mammary gland^38^. Similarly, the FGF signaling pathway is required for the formation of homologous tubular pouches in the sea urchin, a related echinoderm, suggesting that these mechanisms are conserved within this clade^30^. These similarities with vertebrate morphogenesis suggest that the coupling of proliferation with cell migration may be an ancestral deuterostome innovation for the formation of tubular organs. It is further possible that FGF signaling may be independently important for regulating cell migration. However, given its required role for cell proliferation, without which the tubes do not form, future work will be required to decouple and test these putative functions.

### Distinct signals regulate tube elongation, cell fate and orientation

The shape of epithelial tubes is guided by signaling pathways from the embryonic environment. However, because of the great morphological complexity of tubular organs, our knowledge on the contribution of the extrinsic cues to distinct steps of tubular organ establishment is still limited^29^. The sea star hydro-vascular organ offers a simplified system in which we defined how the Delta/Notch, Wnt and FGF pathways each uniquely contribute to distinct aspects of tubulogenesis (Fig. 8c).

Tube cells share a common origin with muscles, as both tissues derive from the mesoderm^22,23,30^. Here we report that when we block Delta/Notch signaling, an important regulator of mesodermal specification^39^, excess muscle fibers enwrap the tubes causing constriction (Fig. 4e–g). There are two possible hypotheses on the origins of these fibers. First, Delta/Notch signaling acts on cells from the adjacent esophageal muscles to induce migration to the tubes and differentiation once there. Alternatively, our data support that Delta/Notch signaling might inhibit epithelia tube cell transfating into a muscle-like phenotype. Notch inhibits skeletal muscle differentiation in many contexts, including during vascular system differentiation^40^. Furthermore, the observation that tubes are shorter when Delta/Notch pathway is blocked might be a consequence of cells transfating into muscles, therefore leaving the epithelium with fewer cells. From our data, these ectopic muscle fibers appear to originate from tube epithelial cells (Fig. 4f, g). Moreover, we observed a few cells from the tubes undergoing EMT to become mesenchymal (cell 1 in Fig. 2b and Supplementary Movies 3, 6), suggesting that some cells retained the ability to acquire a distinct mesodermal fate. This phenotypic change, that eventually leads to loss of epithelial function, has been observed to be the cause of lung fibrosis^41^, but it is unclear what pathways guide it. Overall, our data in the sea star suggests that the Delta/Notch signaling might be responsible for such phenotypic change in complex organs.

Although correct orientation is critical for tube function, very little is known about how tubular organs orient within the growing embryo^29^. Taking advantage of the simple morphology of the sea star tubes, we found that the Wnt signaling through the Frizzled 1/2/7 receptor regulates tube orientation. Frizzled 2 receptor controls branch formation and shape of the epithelial tube of the highly branched mouse lungs^42^, but a clear link with the overall orientation of this structure was not established. A question raised by these findings is the mechanism through which Fz1/2/7 affects orientation of tube growth. We hypothesize that Wnt signaling may be acting through the planar cell polarity pathway rather than the canonical pathway to guide shape changes and tube directionality. In support of this premise, Wnt signaling has been found to coordinate cell polarity in groups of cells, for instance by controlled cell division orientation in the mouse stratified epithelium^43^. Another hypothesis to consider is that activation of Fz1/2/7 drives expression of membrane proteins that bind to ECM components to properly orient the tube outgrowth directionality. Last, the fact that the esophagus and the tubes, both expressing Fz1/2/7, were shorter than controls but kept the same length ratio raises the possibility that Wnt signaling also stimulates cell proliferation in these tissues by stimulating cyclin genes, as has been shown in the pancreas^44^.

These findings all highlight the conserved importance of Wnt signaling in defining the shape and orientation of complex organs across evolution. Importantly, our study adds that Wnt signaling specifically guides the 3D orientation of the growing tube in embryogenesis.

### FGF target genes reveal a breadth of mechanistic insight

Unraveling the downstream targets of the FGF signaling pathway, and understanding the conservation of molecular pathways in different models, are critical objectives that need to be addressed in tubulogenesis^29^. A unique aspect of our study is that we found FGF perturbation to selectively ablate the tubes, allowing us to use RNAseq to discover putative transcriptional targets of the FGF pathway. In the brittle star *Ophiura filiformi*, another echinoderm, a similar RNAseq study of embryos without functional FGFR showed a lack of skeleton formation^32^. As in the sea star hydrovascular organ, *Alx4*, *Six1/2* and *MHC* were downregulated when FGFR was blocked, though other targets like the transporters and the ECM remodeling enzymes were not affected. Indeed, the most abundant candidates we found in our analysis were metalloproteases and tissue inhibitors of metalloproteases (expression examples for *matrixin* and *FcoII*, Fig. 7b–d). Similarly, a recent study found that tube cell precursors are enriched in genes related to the extracellular matrix and adhesion^45^. Dynamic remodeling of the extracellular matrix components (ECM) at the basal and apical compartments of tubes is a fundamental factor in tube shape and growth^46^. For instance, the basal lamina controls the size of the Drosophila tracheal tubes^47^, and remodeling of the ECM through metalloproteases is essential for the elongation of the sea urchin larva skeleton, a syncytial tube^48^. We showed that the metalloprotease inhibitor FcoII contributes to remodeling of the basal lamina at the anterior end of the growing tubes (Supplementary Fig. 9a), and both matrixin and FcoII proteins have a signal peptide, suggesting they are secreted (Supplementary Figs. 7 and 8). However, further studies should address the mechanism used by the metalloprotease and its inhibitors to contribute to ECM remodeling around the growing tube. In many systems, the balance of these factors is regulated by the FGF pathway.

FGF is essential for myotube guidance in fly muscles, and for muscle specification in sea urchin larva^30,49^. Also in the sea star system another group of FGF downstream candidates were muscle genes, and we focused on the expression of the terminal differentiation gene MHC since it is highly abundant. We discovered two populations of muscles not previously described in the sea star larva: muscles in the hydropore canal and longitudinal muscle fibers along the tubes (Fig. 7f–j). The longitudinal muscle allows for the tubular organ to contract (Supplementary Movie 11), while the movements of cilia inside the lumen helps moving fluids or particles in the tubes (Supplementary Movie 4). Studies in other echinoderms showed that larvae have smooth muscles^30^, suggesting this is also the case of the longitudinal muscles around the hydro-vascular system. Our findings show that the hydro-vascular organ also includes muscles that surround this organ in a way that resembles the vasculature or the intestine, specialized tubes that can autonomously contract to move their contents forward.

### A conserved regulatory network for branching morphogenesis

In our RNAseq analysis of FGF targets, we identified the transcription factor *Six1/2*, which is selectively expressed in the hydropore canal (Fig. 7l, m). By Cas9 knockout, we found it to be essential and selective for forming the hydropore canal branch point (Fig. 7n, o). In earlier stages, Six1/2 was found to be expressed in the mesodermal tube precursor cells in both sea stars and sea urchins^18,22,30^. In an example of potentially homologous function, in the mouse, Six1 plays a critical role in a subpopulation of collecting tubule epithelial cells in the developing kidney^50^. *Six1/2* is also expressed by distal epithelial tips of branching tubules in embryonic lungs where it coordinates FGF signaling^51^. These important parallels between formation of the branch point in the sea star hydro-vascular organ and different examples in mice suggest that Six1/2 activation by FGF may be a conserved node in the regulation of branching morphogenesis. An advantage of the sea star system is that while mammalian organs have multiple branches, the sea star tubes offer a simple system with a single defined branched point observable in vivo (Fig. 8d). This system will enable future studies to unravel what aspects of branching are specifically regulated by Six1/2 and its interactions with FGF signaling.

### The hydro-vascular organ offers insight into organ evolution

Our results support the idea that the basal toolkit to make vertebrate tubular organs was already established at the root of the deuterostome clade, the group that includes vertebrates. We revealed that, as in vertebrate organs, tubulogenesis involves cell migration coupled with proliferation in the sea star hydro-vascular organ. Like in vertebrate organs, proliferation happens in growth zones, especially at the tube tips, and it is regulated by FGF signaling. Six1/2 is a conserved target of FGF signaling that is critical for establishing branch points. Given the simplified morphology of the sea star tubes compared to highly branched vertebrate organs, and the technical advantages that the system offers for in vivo studies, we were able to discover additional roles of signaling pathways in tubulogenesis. We propose that the development of the sea star hydro-vascular organ provides important insight into the fundamental evolutionary steps that preceded the appearance of the more complex vertebrate organs.

## Methods

### Embryo and larva cultures

*Patiria miniata* adult animals were obtained from Pete Halmay (peterhalmay@gmail.com) and Marinus Scientific (info@marinusscientific.com) and kept at 15 °C in a sea water aquaria. Gonads were surgically obtained by small cuts on the oral side. Embryos and larvae were cultured at 16 °C. Oocytes were microinjected, then treated with 1-methyladenine (Acros Organics) at a final concentration of 10 μM to induce meiotic resumption. Adult sea stars do not show any phenotypic trait that can differentiate among individuals; therefore, animals were randomly chosen. The sex of the larvae is always unknown and individual larvae with the same genotype were identical, therefore allocation in experimental groups was not relevant in our study and sex of the larvae was not considered. Animals are collected from the ocean and it is not possible to assess age. Sea stars are invertebrate and do not require prior ethical approval.

### Plasmid constructs and RNA in vitro transcription

The H2B-GFP construct was amplified from first strand cDNA reverse transcribed from total ovary mRNA and then cloned into pCS2 + 8 as C-terminal GFP fusions^52^. LifeAct-mcherry construct was a gift from Cynthia Bradham. Both plasmids were linearized with NotI to obtain a linear template DNA. mRNA was transcribed in vitro with the mMessage mMachine SP6 followed by the polyadenylation kit (Life Technologies, #AM1340), then purified using lithium chloride. In vitro transcribed mRNA was injected at a final concentration of 500 ng/ul for each marker. Prophase arrested oocytes were injected with ~20 picoliters of H2B mRNA.

### Perturbation experiments with CRISPR and Cas9 MASO injection

Guide RNAs (gRNAs) were designed with the online software www.crisprscan.org and purchased from Synthego. gRNAs stock concentration was100pmol/μl each. The Cas9 plasmid used was pCS2-3xFLAG-NLS-SpCas9-NLS (Yonglong Chen, Addgene plasmid #51307). The plasmid was linearized with NotI, mRNA was transcribed with SP6 polymerase and stored in nuclease-free dH2O. For each knock out experiment, injection solutions had Cas9 mRNA at a final concentration of 750 ng/μl and a mixture of 2–3 gRNAs to target genomic DNA at a final concentration of 150 ng/μl each. Control larvae were injected with Cas9 mRNA only. Embryos were kept at 18 °C. Guide RNAs sequences are in Supplementary Data 1. Genomic DNA of individual larvae (*n* = 3–6) was sequenced to test for CRISPR Cas9 induced mutations as previously described^53^. Genomic sequences used to find *Patiria miniata* genes and to design gRNAs were analyzed with the resources at echinobase.org^54^. The frequency of mutations in each larva was 100%, every single larva sequenced had mutations.

A MASO (GeneTools, Philomath, OR) complementary to *Pm-Six1/2* transcript 5′ UTR (5’-CGTGAAGCCAAACGACGGCAACATG-3’) and a MASO complementary *to Pm-FcoII* (5’-GTTAATTCACGTCCT-3’) were used at a final concentration of 800 μM each to block protein translation. A scrambled MASO sequence was used as control. Prophase arrested oocytes were injected with 20 picoliters of morpholino antisense oligonucleotide (MASO) or Cas9+gRNAs solutions in nuclease free water.

### Individual Larva genotyping and TIDE Spectral Decay Analyses

Individual larvae were genotyped using PCR primers and the resulting amplicons specific to each targeted region (Supplementary Data 2). All individual mutant embryo PCRs were sequenced. For mutagenesis analysis: sequence trace files (.ab1) for CRISPR-Cas9 edited and Cas9 injected control larvae were retrieved following sequencing performed through Azenta Genewiz (https://www.genewiz.com/). For all genotypes relevant, Cas9/sgRNA mutation penetrance and distribution of mutation types were analyzed using differential sequence-trace chromatograph analysis with two different analytical packages. The first package: TIDE (Tracking of Indels by Decomposition (V. 3.3.0);^55^ is used for the Six1/2 and Delta mutants. For larger indels, SynthegoICE (V.3) was used (Inference of CRISPR Editing; ice.synthego.com^56^. Total Efficiency, corresponding to the fraction of mutant sequences detected is displayed on each spectra for CRISPR mutants (Supplementary Fig.11). Each of the results show complete penetrance of the Cas9 mutations, that is, each larvae showing the mutant phenotype had corresponding genomic mutations, within the target site, and each larva with a genomic mutation shown a mutant phenotype. The mutations also showed broad size and distribution of mutations as expected in an F0 mutagenesis. An estimated percentage of mosaicism is quantified from spectral data and is shown in the supplement (Supplementary Fig.11).

### Live imaging, single nucleus tracking and statistical analyses

For time-lapse recordings, larvae were embedded in 1% low melting point agarose (Sigma), covered with sea water, mounted on a MatTek 35 mm glass bottom dish, and imaged at room temperature (22 °C). For tube elongation movies, larvae of two independent experiments expressing H2B-GFP were imaged for 2 days capturing z-stacks with 3 μm step size every 5 min. For each experiment, both the left and the right tubes were imaged, and nuclei were tracked. Movies and images of fix samples were taken on a Nikon Yokogawa W1 spinning disk microscope (NIS Elements version 5.21.03) with 40× silicon immersion oil objective. Images were processed and analyzed in Fiji (https://fiji.sc/ lmageJ 1.53c) using the manual tracking plugin (Fabrice Cordelières, Institut Curie, Orsay). To track the displacement of individual nuclei, we first normalized the movies by defining the starting point of tube elongation (*t* = 0) as the tubes were 50 ± 5 μm long, and the final point of tracking (*t* = 40–45 h) as the tubes were 80 ± 10 μm long. Only nuclei of the tube epithelial cells that were in focus and moving along the *x* and *y* axes were tracked. Bright field movies were taken with a Zeiss Axio Vert.A1 (Zen 2.5 Blue Edition version 2.5.75.0) in continuous mode. DIC Supplementary Movie of cilia moving in the hydro-vascular organ (Supplementary Movie 4) was taken on an Olympus FV3000 confocal microscope (Olympus Fluoview V4.2, Brown University Leduc Facility). Student’s t-test was performed to assess statistical significance, values of p < 0.001 are represented as ****. Graphs and statistical analyses were performed using Prism (GraphPad Software, V.7.0.0). Rose plots were made with GeoRose (https://www.yongtechnology.com V.0.5.1.1). Figures were made with Adobe Illustrator (V.27.2).

### EdU incorporation and pharmacological perturbations

5-ethynyl-2-deoxyuridine (EdU) pulse labeling was performed with the Click-IT EdU imaging kit (Life Technologies; Cat.#:C10340) as described in^57^. A final concentration of 10 mM EdU in sea water for 30 min was used. 25 to 50 tubes were analyzed for each stage. The Cdk4/6 inhibitor Abemaciclib (LY2835219, Selleckchem Cat.#S5716)^21^ was added to larval cultures at 16 °C at a final concentration of 10μM. The γ-secretase inhibitor DAPT (N-[N-(3,5-difluorophenacetyl)-L-alanyl]-S-phenylglycine t-butyl ester, FisherScientific, Cat.#J65864.MA)^39^ was added to a final concentration of 10 μM. To perturb the Wnt pathway, the small molecule porcupine inhibitor ETC-159^27^ was used at a final concentration of 5 μM (Selleckchem, Cat.#S6616). The FGFR inhibitor SU5402 was used at 40 μM final concentration (Sigma, Cat.# SML0443) and the MEK inhibitor U0126 at 10 μM (Selleckchem, Cat.#S1102). Beta-aminopropionitrile (BAPN, Sigma Cat.#A3134) was used at a final concentration of 300 μM in sea water. All other drugs were dissolved in DMSO and the optimal drug concentration was chosen after testing concentration ranges from 0.1 to 100 μM. All drugs were added at the end of gastrulation, prior to the development of the hydro-vascular organ tubes. A corresponding volume of DMSO was added to sea water in controls. Experiments were performed in three independent biological replicates.

### Fluorescent in situ hybridization (FISH) and immunofluorescence

For fluorescence in situ hybridization (FISH), larvae previously fixed in 4% paraformaldehyde were hybridized for 1 week with each digoxigenin-labeled riboprobe that were transcribed from linearized DNA (list of primers in Supplementary Data 3) with the DIG-RNA labeling kit (Sigma-Aldrich, Cat:#11175025910). Signal was detected with an anti-DIG antibody (Roche Cat:#11207733) diluted 1:2000 following the protocol of the tyramide signal amplification kit (PerkinElmer, Cat:#NEL760) as shown in ref. ^58^. For immunofluorescence experiments, larvae were fixed in 2% PFA in PBS for 20 min at RT followed by 10 min in 100% methanol (this step was skipped only for experiments where phalloidin was added). To image laminin together with phalloidin the methanol step was skipped, resulting in a less sharp laminin staining. Larvae were then washed in PBST (0.1% Tween20) and incubated overnight at 4 °C with 1 mg/ml BSA and 4% sheep serum in PBST and one or more of the following: Alexa-fluor 488 Phalloidin 1:300 (Molecular Probes, Cat.#A12379), anti-beta tubulin antibody 1:100 (Hybridoma bank, Cat.#E7), anti-laminin antibody 1:300 (Abcam, Cat.#AB11575), monoclonal Anti-MAP Kinase, Activated (Diphosphorylated ERK-1&2) pERK 1:100 (Millipore Sigma, Cat.#M8159). Samples were then washed three times with PBST and incubated with the corresponding AlexaFluo secondary antibodies (Invitrogen anti-mouse Cat.#A11001 and anti-rabbit Cat.#A-11037) at a final concentration of 1:2000 for 2 h RT. Nuclei were labeled with DAPI 1 mg/mL (Thermo Fisher Scientific, Cat:#D3571). Samples were imaged on a Nikon Yokogawa W1 spinning disk microscope (NIS Elements version 5.21.03) and processed in Fiji (https://fiji.sc/ lmageJ 1.53c).

### RNA extraction and qPCR

RNA from 100 larvae was isolated with the RNeasy Micro kit (Qiagen, Cat#:74004). cDNA synthesis was performed using the Maxima kit (Life Technologies, Cat#:K1641). qPCR was performed with Maxima SYBR master mix (Life Technologies, Cat#:FERK0222) using ABI7900 and QuantStudio3 (ThermoFisher, V2.6.0) real-time PCR systems. Transcripts were normalized to ubiquitin and qPCR data were analyzed using the delta-delta CT method. Three biological replicates and three technical replicates were performed. A threshold of twofold difference was chosen as a biologically meaningful change. Points on the graphs show the mean of the three technical replicates and error bars represent biological replicates.

### Differential RNAseq

Total RNA was isolated from 3-day-old larvae (LG) stored in Trizol (Life Technology, Cat#:15596-026) in three biological replicates. Samples were sent for RNA extraction, library preparation, Illumina HiSeq 2 × 150 bp sequencing, quality control and RNA-Seq data analysis to Genewiz (www.genewiz.com). Differentially expressed genes with relative FDR and GO term results (Interproscan V.5.38-76.0) are provided in Supplementary Data 4.

### Statistics and reproducibility

For all experiment multiple factors were analyzed to test the significance of the results. Sample size was chosen based on the number of embryos sufficient to perform statistical student t-test, around 15–50 sea star embryos for each experiment that were repeated in 3–5 biologically independent experiments. Statistical significance was assessed with two-sided Student’s t-test (unequal variance, ****p ≤ 0.0001, ***p ≤ 0.0005, **p ≤ 0.005, *p ≤ 0.05, ns= not significant). Samples number and p values are indicated in the figure or figure legend. Graphs were created in Prism. All attempts at replication were successful. No data were excluded from the analyses. All sea star embyos used for these experiments derive from gonads of at least three different females and males, therefore in every experiment there is a mix of genotypes that was randomly chosen. To control for bias, for each genotype we counted the phenotype and performed precise measurements with statistical tests. Being only one researcher performing all experiments the investigator was not blinded to allocation during experiments, but the outcome assessment was performed with all authors.

### Reporting summary

Further information on research design is available in the Nature Portfolio Reporting Summary linked to this article.

## Supplementary information

Supplementary information

Description of Additional Supplementary Files

Supplementary Data 1

Supplementary Data 2

Supplementary Data 3

Supplementary Data 4

Supplementary Movie 1

Supplementary Movie 2

Supplementary Movie 3

Supplementary Movie 4

Supplementary Movie 5

Supplementary Movie 6

Supplementary Movie 7

Supplementary Movie 8

Supplementary Movie 9

Supplementary Movie 10

Supplementary Movie 11

Reporting Summary

## Data Availability

The RNA-seq data generated in this study have been deposited in the NCBI BioProject database under accession code PRJNA898435. The processed RNA-seq data and the GO term analysis results are provided as Supplementary Data 4. The Supplementary Movies in Figs. 2 and 3 analyzed in this study are provided as Supplementary Movies 5, 6, 7, 8. The Supplementary Movie used for images in Fig. 1g is provided in Supplementary Movie 4; the Supplementary Movie used for Fig. 2i is provided in Supplementary Movie 9; the video used to make Fig. 3g, h is provided in Supplementary Movie 10. Source data are provided with this paper.

## Source data

Source Data
