# Supplementary material for: Molecular mechanisms of tubulogenesis revealed in the sea star hydro-vascular organ
Source: Nat Commun. 2023 May 9;14:2402. doi: 10.1038/s41467-023-37947-2 (PMC10170166; doi:10.1038/s41467-023-37947-2)
Supplement: Supplementary file 1 — Supplementary information [file 41467_2023_37947_MOESM1_ESM.pdf]

Molecular mechanisms of tubulogenesis revealed in the sea star hydro-vascular organ.

Margherita Perillo, S. Zachary Swartz, Cosmo Pieplow, Gary M. Wessel

Correspondence to: mperillo@mbl.edu (MP); rhet@brown.edu (GW)

This PDF file includes: Supplementary Figures 1-15

Supplementary Figures

Sea star stages	Developmental timing (15-18°C)	Hydro-vascular organ naming	Features
**Gastrula (G)** 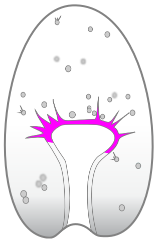	44-48 hpf	Precursor cells (mesoderm)	Hydro-vascular organ precursor cells are located on the tip of the growing gut and are polarized.
**Late Gastrula (LG)** 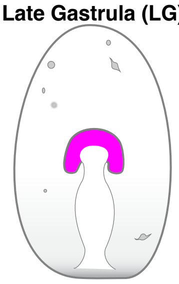	60-72 hpf	Tubes	Tubulogenesis starts. From the tip of the gut the precursor cells start to migrate on the sides of the gut. Two tubes grow towards the posterior end of the larva.
**Early Larva (EL)** 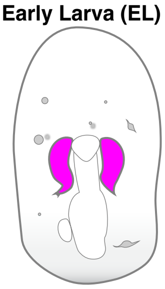	4d	Tubes	Two bilateral tubes are formed on the left and right sides of the gut. Formation of the hydropore canal, the only opening towards the outside environment.
**Larva (L)** 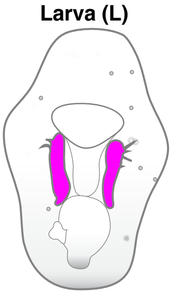	5-7d	Tubes	Tubes elongate. This stage last 3 days that we defined as L1 (5d), L2 (6d), L3 (7d) each subsequent day of the larva stage
**Late Larva (LL)** 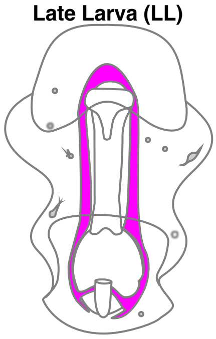	>1w	Hydro-vascular organ	The two tubes merge to form the hydro-vascular organ, a continous tube that has one opening towards the outside environment, the hydropore canal.

Supplementary Fig. 1: Tubulogenesis stages in the hydro-vascular organ. Summary of the formation of the hydro-vascular organ in the sea star Patiria miniata. hpf= hours post fertilization; d= days; w= weeks.

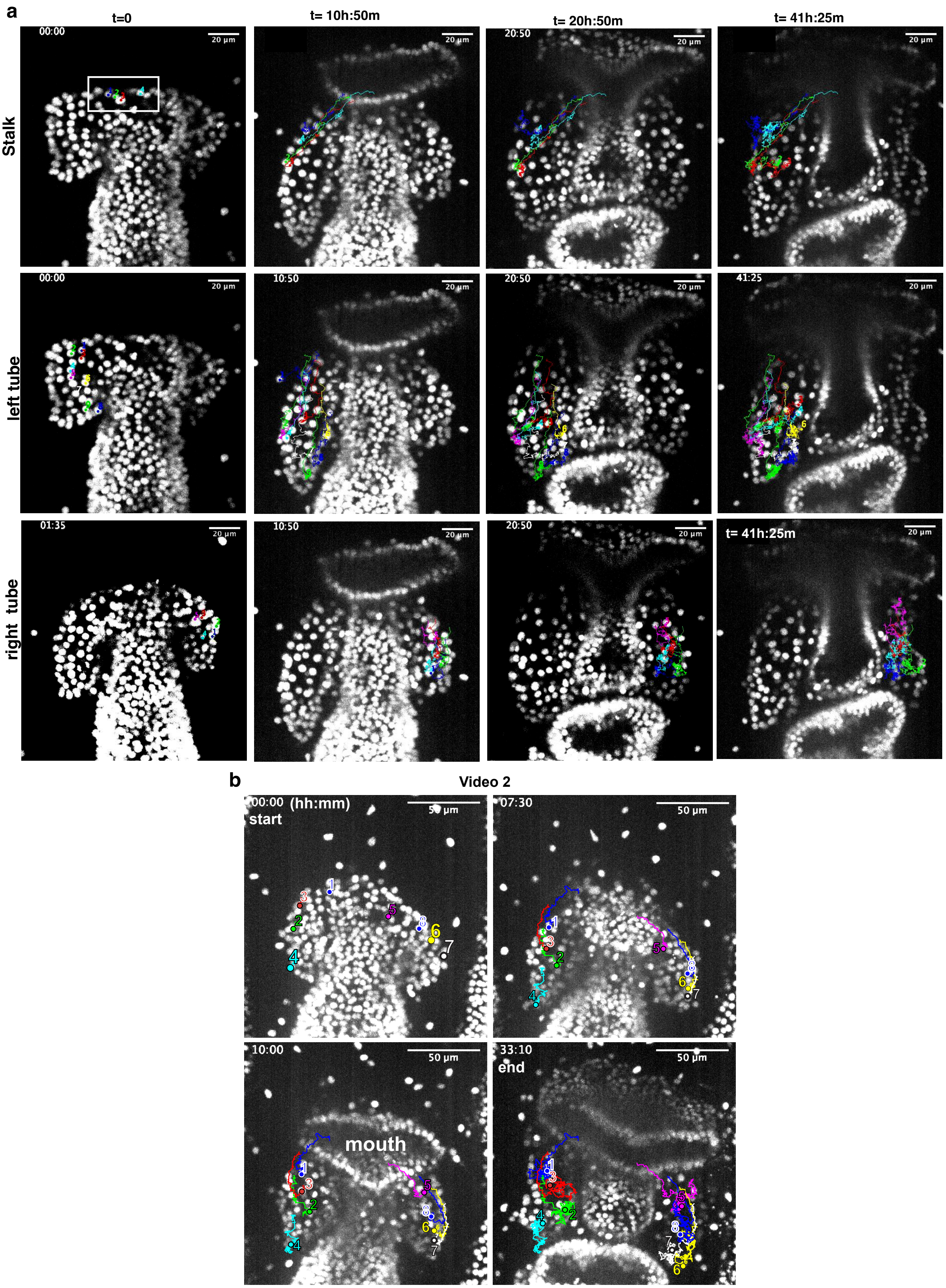

Supplementary Fig. 2: Tubulogenesis involves cell migration. a) Time-lapse images of cell tracking for larva 1 (still images from Supplementary Movie 5, 6 and 7). Tracking of cells of the left tube is shown in main Figure 2 a-d. b) Time-lapse images of cell tracking for larva 2 (still images from Supplementary Movie 8). T=time, h=hours, m=minutes.

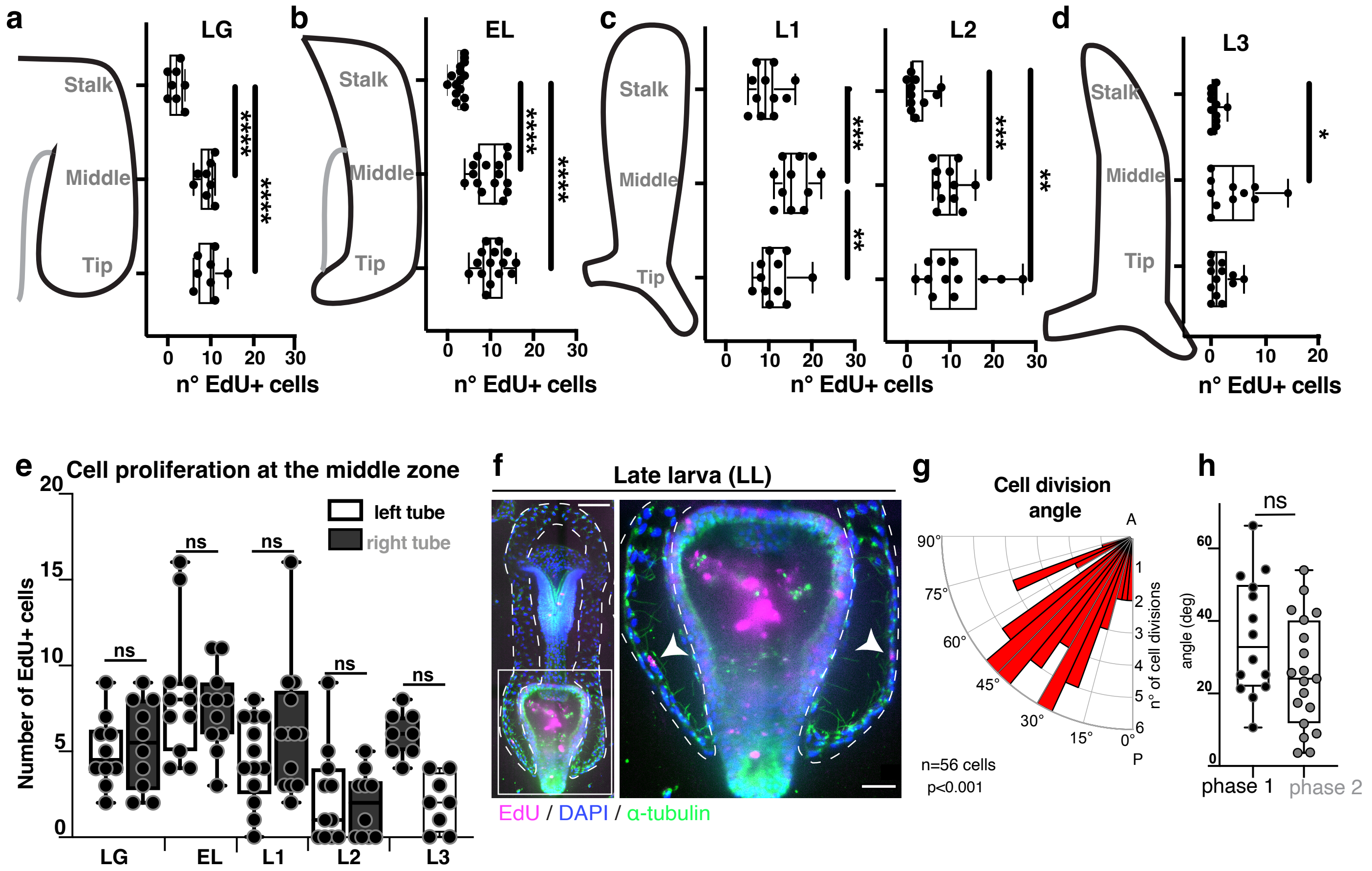

Supplementary Fig. 3: Cell proliferation happens preferentially at the tip of the growing tubes. a-d) EdU positive cells across 3 tube zones: stalk, middle and tip. Graphs represent the data shown in the heat map of Figure 3 e. For a) n=8 biological independent larvae; b) n=15 biological independent larvae. c) n=11 biological independent larvae; L1: ***p=0.0005, **p= 0.0093; L2: ***p=0.0005, **p= 0.0093. d) n=13 biological independent larvae; *p= 0.0425. e) Cell proliferation of the middle zone is the same in left and right tubes. f) EdU incorporation in late larva showing lack of cell division in the tubes, except the left and right somatocoels (the posterior most-end of the tubes). g) Rose plot of cell division angles from 2 movies, Wilcoxon test p<0.0001. h) Cell division angle is the same in the two phases of cell migration. For phase 1 n= 14 and phase 2 n=19 biological independent cells. Statistical significance was assessed by a two- sided Student's t-test (**** p<0.0001, *** p<0.0005 **p≤0.005, *p ≤0.05, ns= not significant). In box plots the median is the middle line, box represents 25th and 75th percentiles, whiskers indicate the minimum and maximum data range. Source data are provided as a Source Data file.

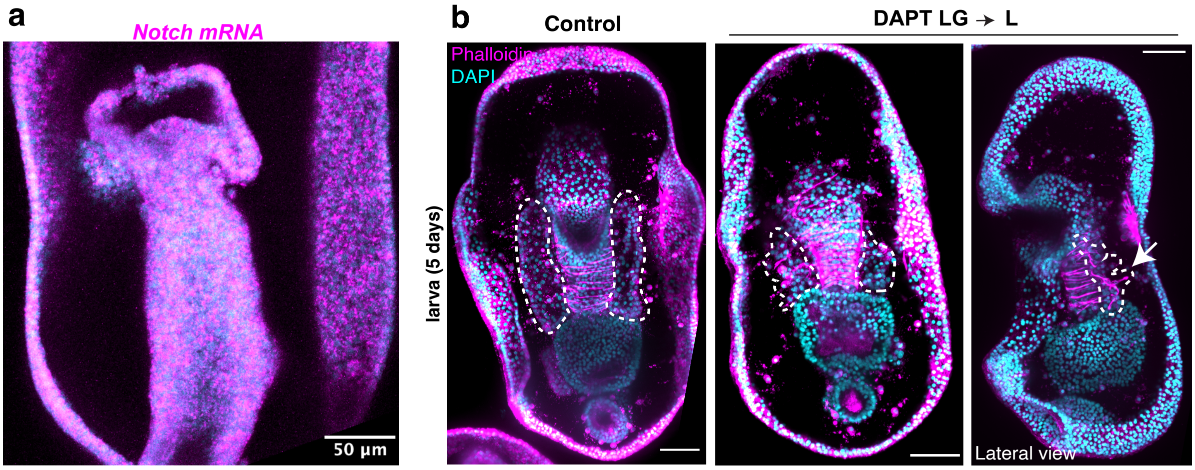

Supplementary Fig. 4: Delta-Notch signaling controls cell fate of tube cells. a) FISH for Notch indicates the gene is ubiquitously expressed. b) Treatments with the Delta-Notch inhibitor DAPT added at the end of gastrulation shows that tubes are shorter but overall larval morphology is intact. All experiments were independently repeated at least 3 times with similar results. Scale bar = 50 µm.

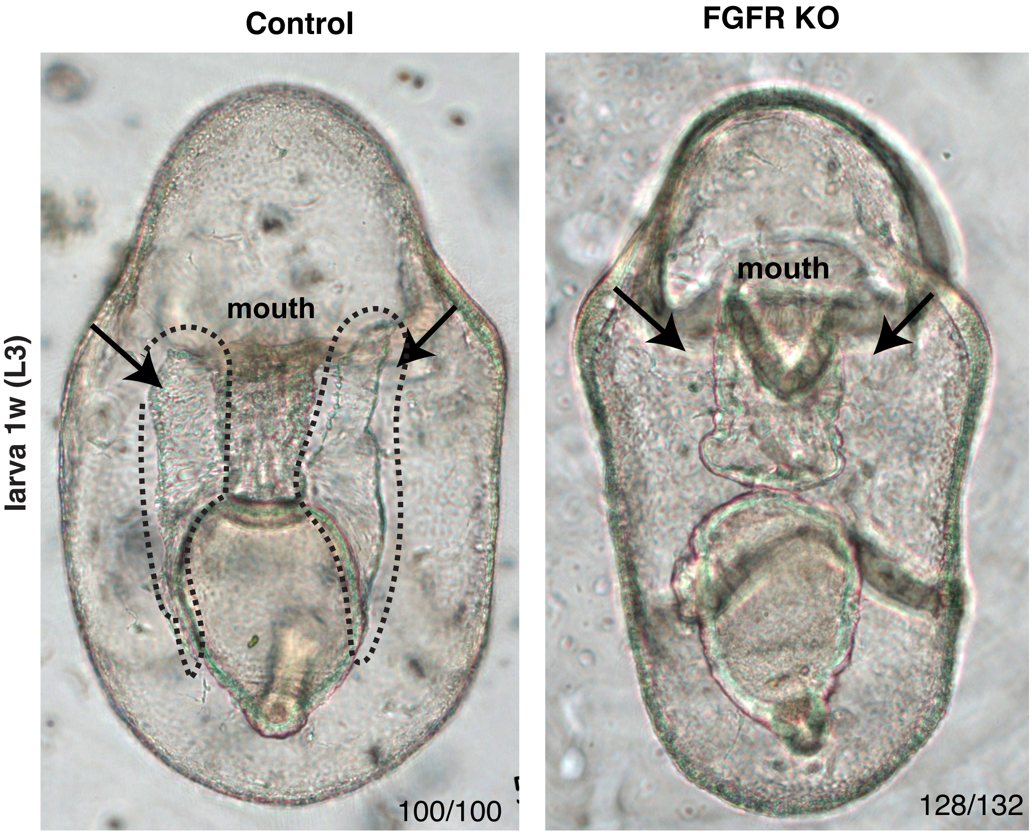

Supplementary Fig. 5: FGFR KO develop normally but lack tubes. Controls larvae and larvae knocked out for FGFR show absence of tubes. w=week. All experiments were independently repeated at least 3 times with similar results.

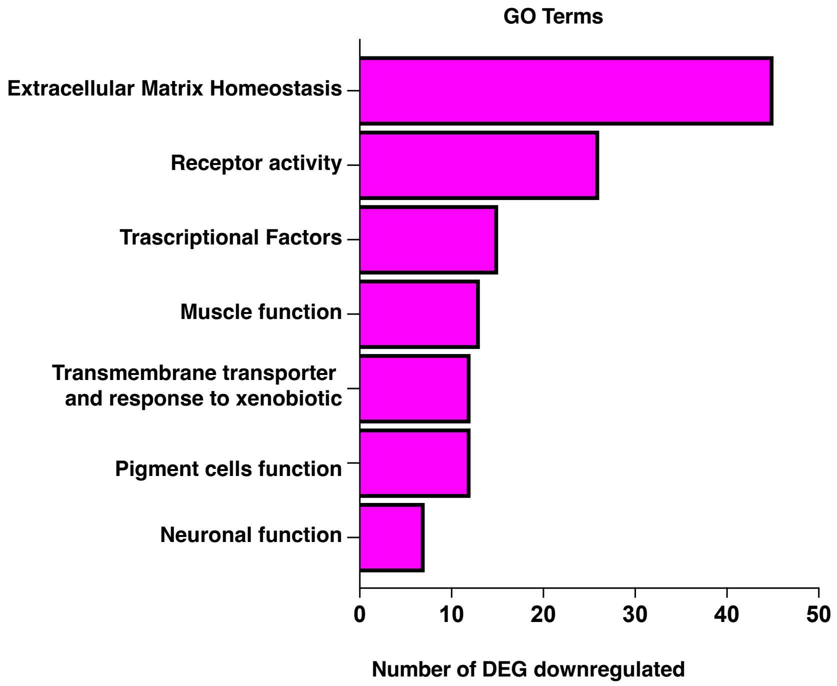

Supplementary Fig. 6: Gene ontology (GO) analysis of RNA-seq candidates. Categories of genes downregulated when FGFR is inhibited. DEG= differentially expressed genes. Source data are provided as a Source Data file.

Pm matrixin signal peptide

matrixin

Prediction: Signal Peptide (Sec/SPI)

Cleavage site between pos. 21 and 22. Probability 0.976835

Protein type	Other	Signal Peptide (Sec/SPI)	Lipoprotein signal peptide (Sec/SPII)	TAT signal peptide (Tat/SPI)	TAT Lipoprotein signal peptide (Tat/SPII)	Pilin-like signal peptide (Sec/SPIII)
**Likelihood**	0.0002	0.9992	0.0001	0.0001	0.0001	0.0001

Download: PNG / EPS / Tabular

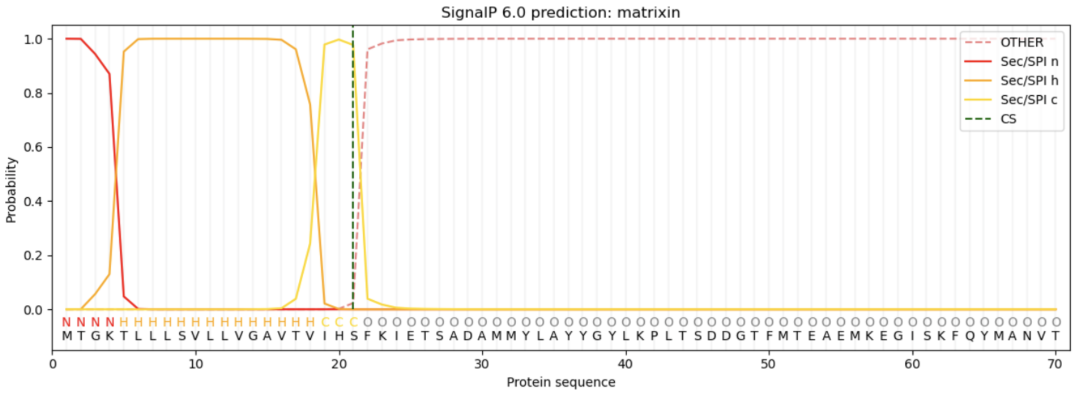

Pm matrixin protein family

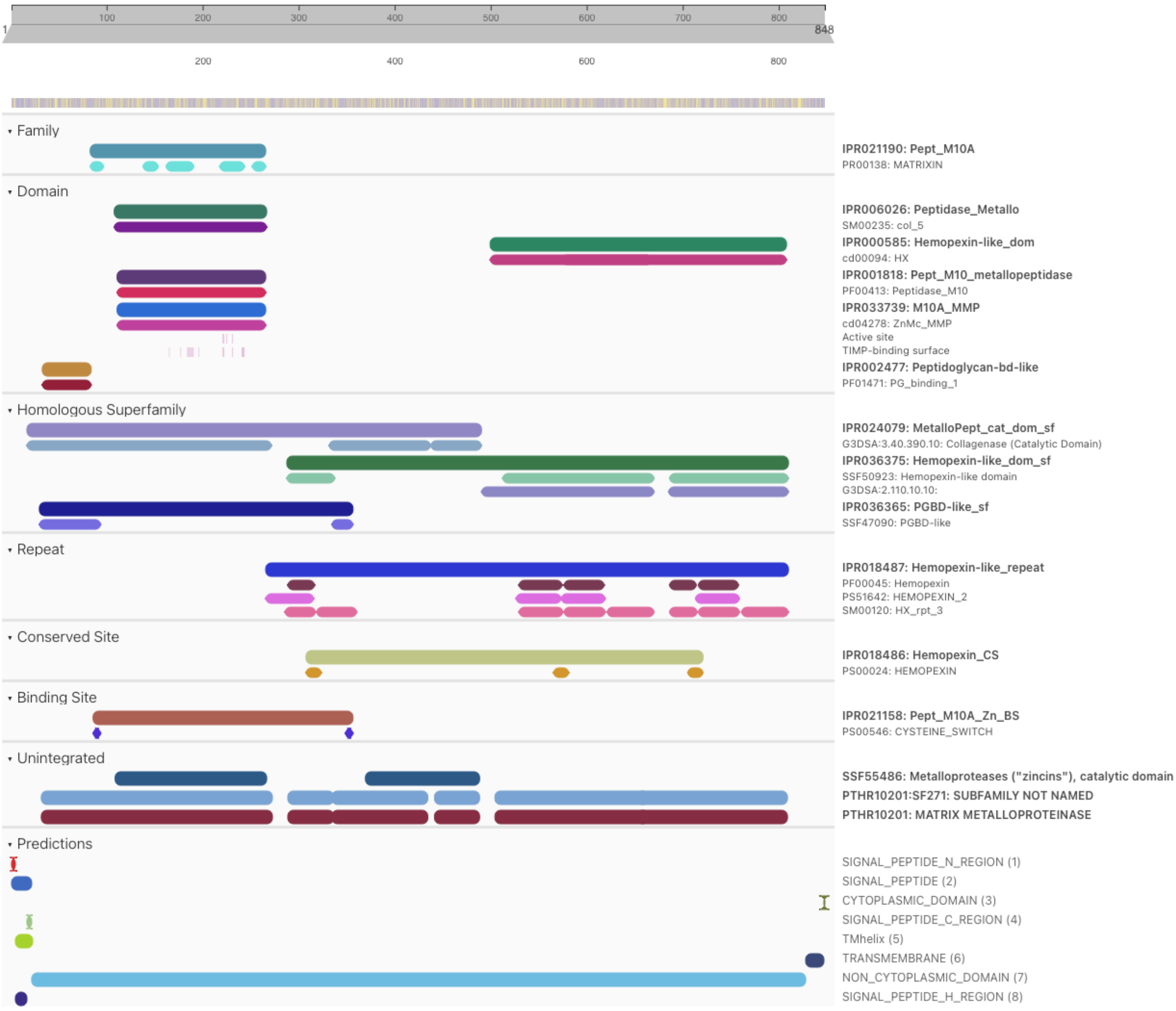

Supplementary Fig. 7: Identification of protein features for matrixin. Signal peptide analysis to identify secreted proteins (SignalP) and Interprot analysis of protein domain to identify protein families.

Pm Fcoll signal peptide

Pm-Fcoll_II_IIIf_7
Prediction: Signal Peptide (Sec/SPI)

Cleavage site between pos. 19 and 20. Probability 0.763446

**Protein type**	**Other**	**Signal Peptide (Sec/SPI)**
**Likelihood**	0.0005	0.9995

Download: PNG / EPS / Tabular

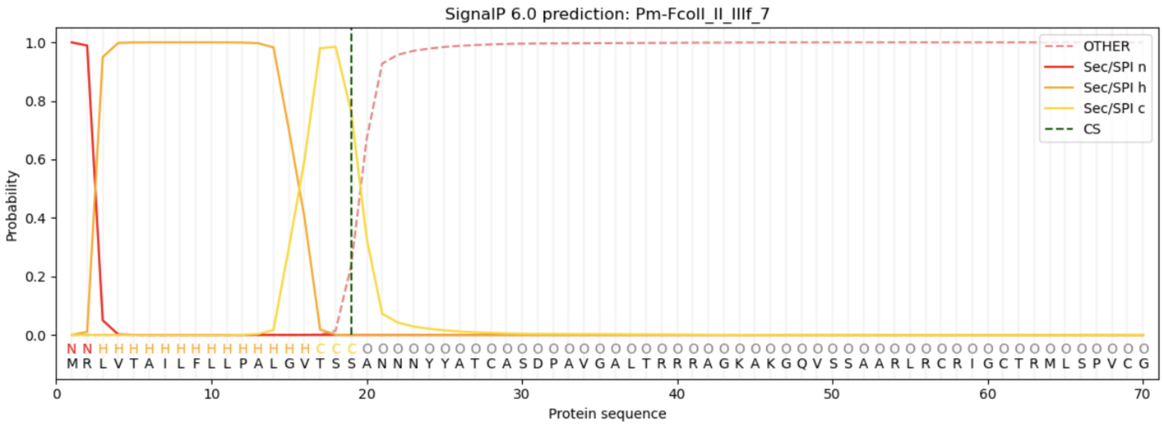

Pm Fcoll protein family

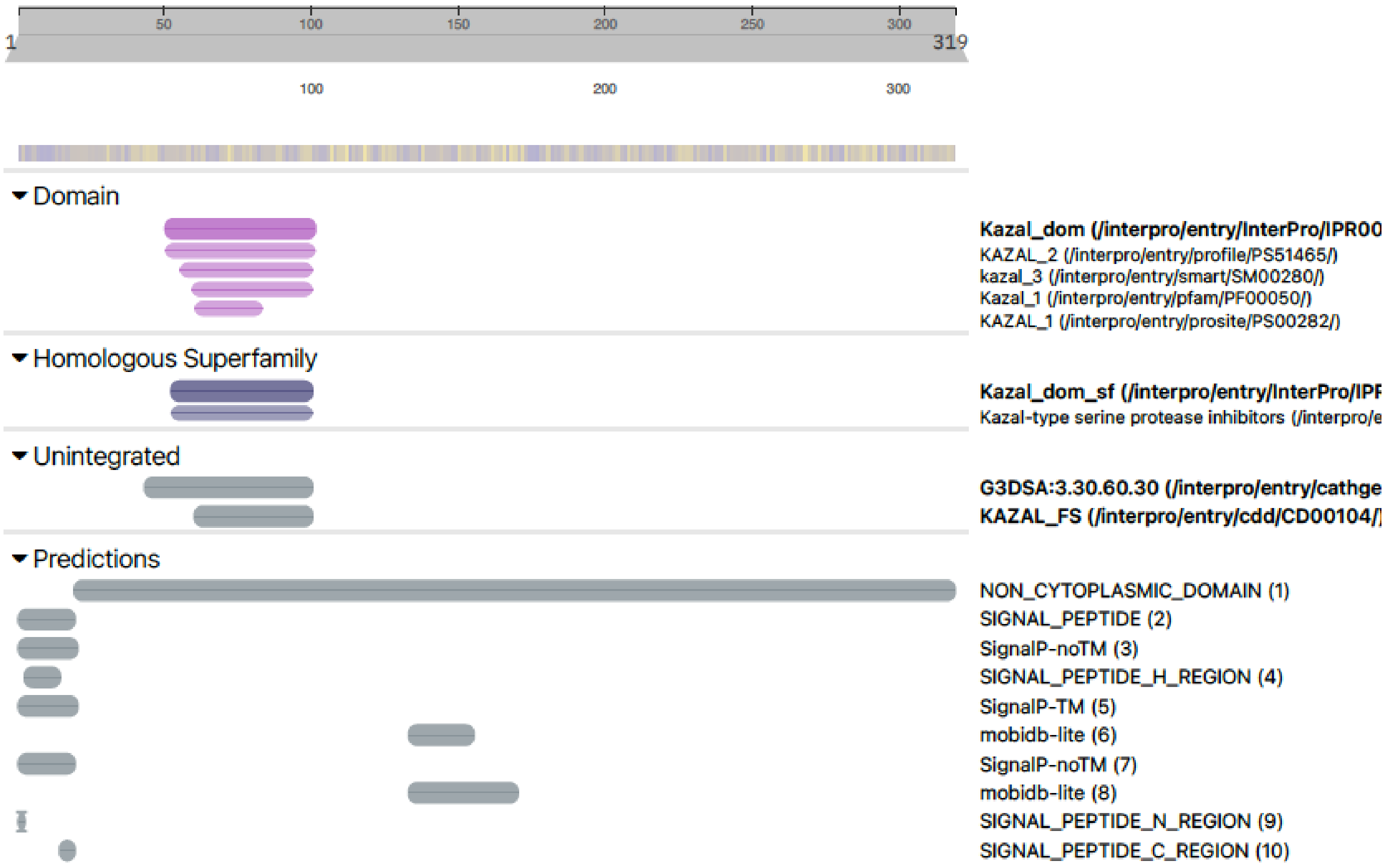

Supplementary Fig. 8: Identification of protein features for FcoII. Signal peptide analysis to identify secreted proteins (SignalP) and Interprot analysis of protein domain to identify protein families.

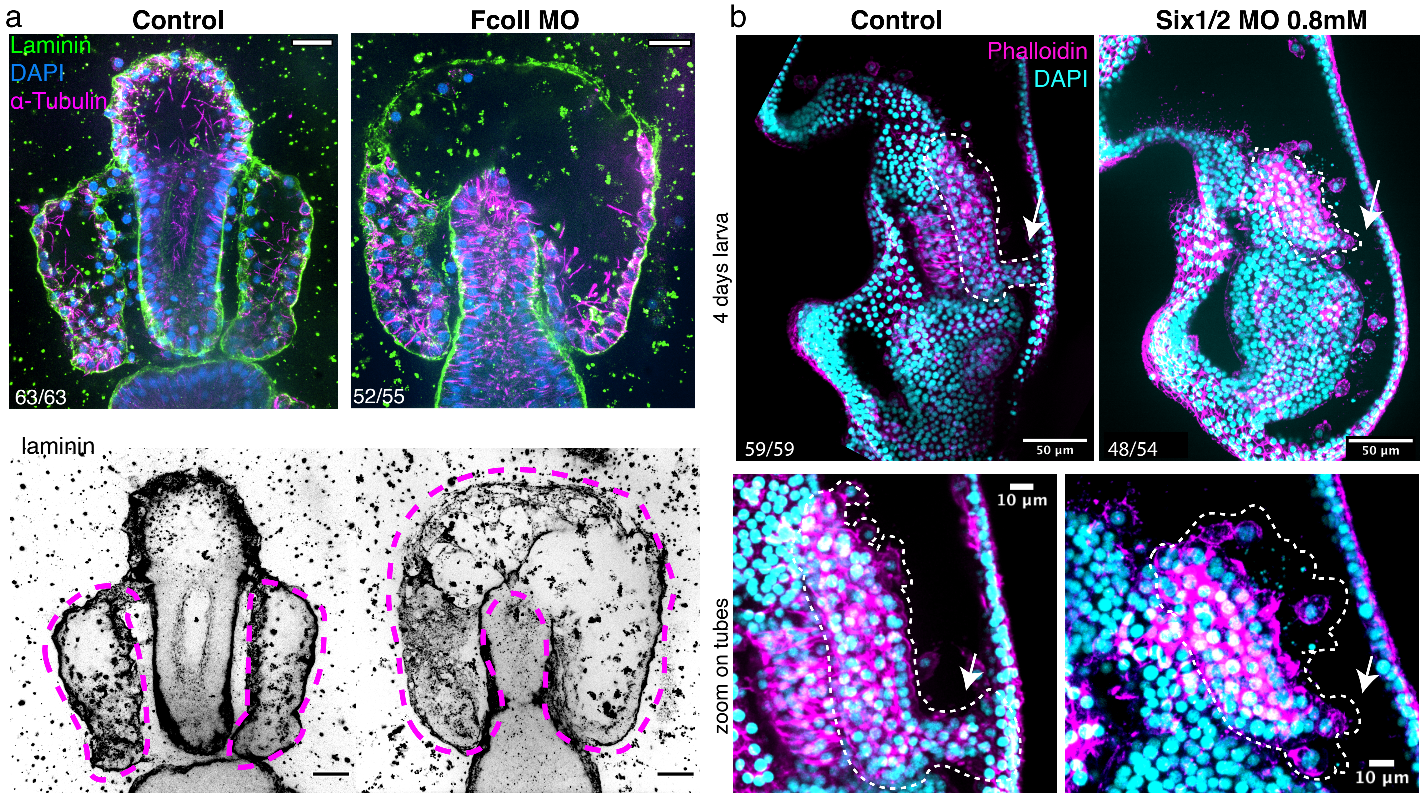

Supplementary Fig. 9: Functional analysis of FGFR targets. a) Larvae knock down with FcoII morpholino (MO) have expanded basal lamina at the anterior side of the tubes. b) Larvae knocked down with Six1/2 morpholino showing lack of hydropore canal. Dotted lines outline the tubes. All experiments were independently repeated at least 3 times with similar results.

Pm Abcc4 protein family

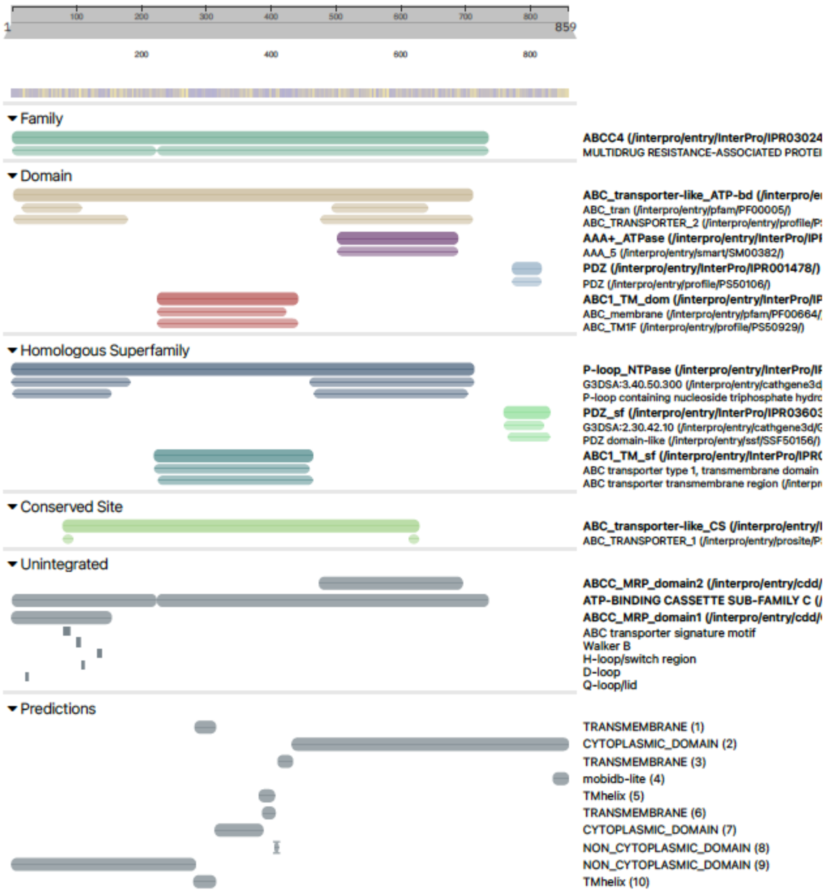

Supplementary Fig. 10: Identification of protein features for Abcc4. Signal peptide analysis to identify secreted proteins (SignalP) and Interprot analysis of protein domain to identify protein families.

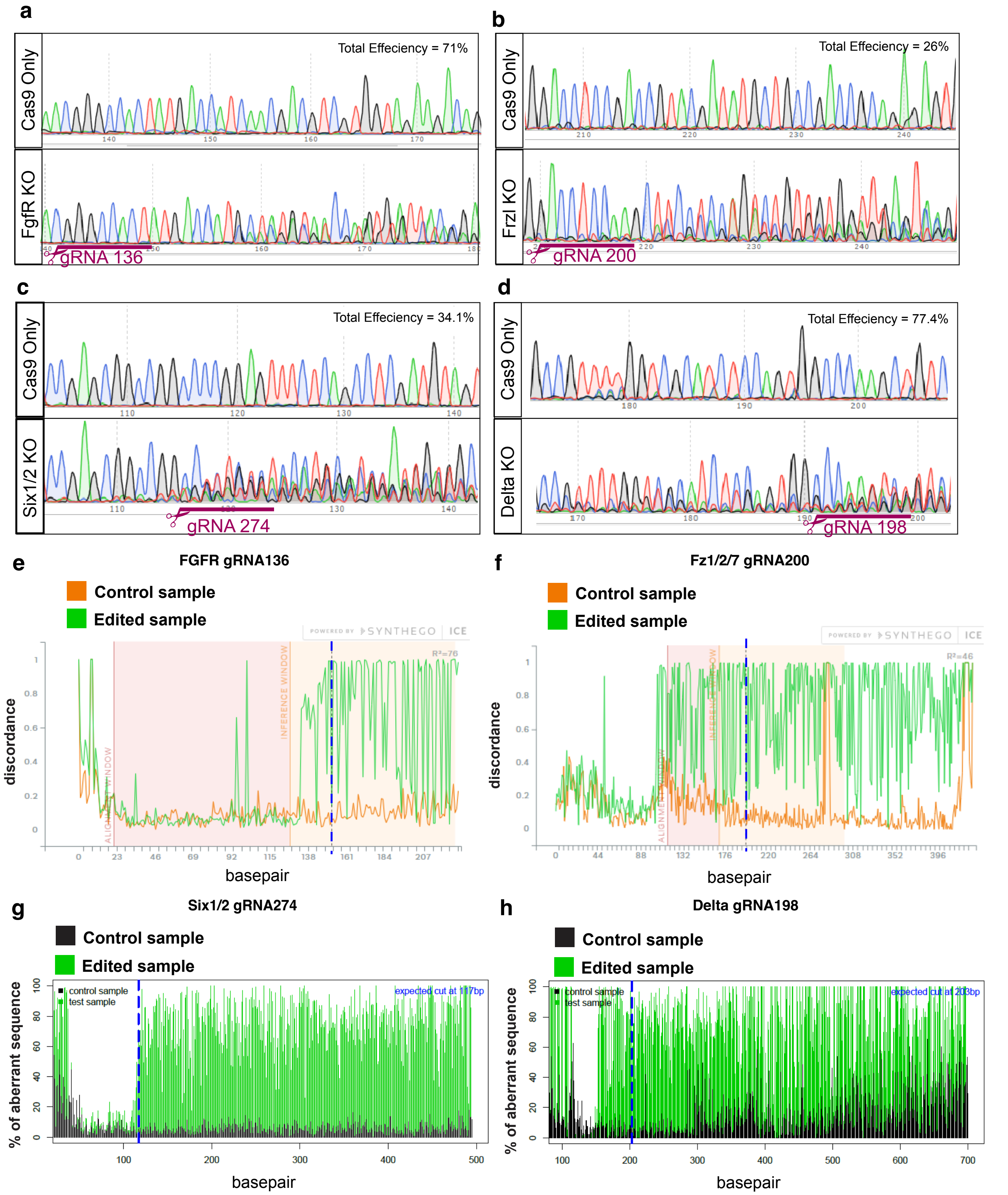

Supplementary Fig. 11: CRISPR Cas9 Mutations. TIDE Analysis of spectral decay for a single representative a) FGFR, b) Fz1/2/7, c) Six1/2 and d) Delta mutant embryos. gRNA sites are shown in magenta, the mutant sequences are aberrant peaks following the cut site. Estimated efficiency is displayed as percentage of total base pairs corresponding to a detected indel for every mutant genotype. Total Efficiency is the percentage of aberrant sequence peaks that are different indel. e-h) Quantification of spectral decay for a single mutant embryo, with the gRNA cut site shown at the respective basepair. Graphs show high % of aberrant sequence, meaning mutants deviate from controls. Blue dotted line indicate sgRNA cut site after wich there is an high aberrant spectra, since CRISPR mutants are inherently mosaic. Note that TIDE package was used for Delta and Six1/2 and SynthegoICE was used for FGFR and Fz1/2/7 since they had large indels that could not be analyzed with the TIDE software (described in the method section).

Delta

```
Delta reference    ATGAGAATTACTACGGACCGACATGTTCCAATATTTGCCGGCCCAAGAACGACAGCTTGG   60
Larva 13           ATGAGAATTACTACGGACCGACGTGTTCCATTNTTTGCCNNCCC-ANAACNANAACTNGG   59

Delta reference    GCCACTGGAGATGCGACCAAAACGGCCAGAAAGTGTGCTTGGATGGCTGGGAGGGAGCCT   120
Larva 13           GCCACTGGNGATGCGACCAAAACGGCCANAAAGTGTGCTTGGATGGCTGGGANGGANCCT   119

Delta reference    TTTGCACCATA  131
Larva 13           TTNNCACCATA  130


Delta reference    ATGAGAATTACTACGGACCGACATGTTCCAATATTTGCCGGCCCAAGAACGACAGCTTGG   60
Larva 14           ATGAGAATTACTACGGACCGACNTGTTCCAATATTTGCCGG-CNAAGAACGACAGCTTGG   59

Delta reference    GCCACTGGAGATGCGACCAAAACGGCCAGAAAGTGTGCTTGGATGG-CTGGGAGGGAGCC   119
Larva 14           GCCACTGGAGATGCGANCAAAACGGNCANAAAGTGTGCTTGGATGGNNNNNNNGNGANCC   119

Delta reference    TTTTGCACCATA  131
Larva 14           TTTNNCACCGTA  131


Delta reference    ATGAGAATTACTACGGACCGACATGTTCCAATATTTGCCGGCCCA----AGAACGACAGC   56
Larva 16           ATGANAATTACTACGGACCGACNTGTTCCAATATTTNNNGGCCCANNANANAAC----NC   56

Delta reference    TT-GGGCCACTGG--AGATGCGACCAAAACGGCCAGAAAGTGTGCTTGGAT--GGCTGGG   111
Larva 16           TTGGGGCCNCTGGGANNATGCGNNCAAAACGGNCAGAAAGTGTGCTTGGATGNGNGTGGG   116

Delta reference    AGGGAGCCTTTTGCACCAT  130
Larva 16           NNGGNGCCTTTTGCACCNT  135
```

gRNA 198 / gRNA2 48

Supplementary Fig.12: Genotype of a single Delta mutant embryo aligned to a representative control embryo genotype sequence. All three gRNA sites are shown in three corresponding colors, with specific deletions highlighted.

Fz1/2/7

```
Fzd1/2/7 reference    CTGACACAATGGCGTTGAGAACCGGAGGAATTTTGCCGGATTTCACGGCCCACACCGTTG  60
Larva 1               CTGACACAATGGCGTTGAGAACCGGAGGAATTTTGCCGGA--------------------  40

Fzd1/2/7 reference    CGATCGTCGTCGTTTTCCTGTGCACACTCTCTGCGGTCGAATGTCAAATCGGCAGCATAT  120
Larva 1               ---------------------------TCTCTGCGGT-----------------------  50

Fzd1/2/7 reference    CGTACGGTCGTTGCGAGCCCATAACCATTCCGCTGTGCCAAGATGTCCCTTACAACGAGA  180
Larva 1               ------------------------------------------------------------  51

Fzd1/2/7 reference    CGATCATGCCGAACTTACTCAACCACAACAGACAGGAGGAAGCCGGCTTGGAGGTCCACC  240
Larva 1               ---------------------------------------------GCTTGGGGGTTCAAC  65


Fzd1/2/7 reference    CTGACACAATGGCGTTGAGAACCGGAGGAATTTTGCCGGATTTCACGGCCCACACCGTTG  60
Larva 2               CTGACACAATGGCGTTGAGAACCGGAGGAATTTTGCCGGA--------------------  40

Fzd1/2/7 reference    CGATCGTCGTCGTTTTCCTGTGCACACTCTCTGCGGTCGAATGTCAAATCGGCAGCATAT  120
Larva 2               --ATGGT----------------------TATGGGCTCG-----CAA--CG-------AC  62

Fzd1/2/7 reference    CGTACGGTCGTTGCGAGCCCATAACCATTCCGC------TGTGC-CAAGATGTCCCTTAC  173
Larva 2               CGTACGAT--ATGC-TGCCGA--------CCGCAGAGAGTGTGCACAGG---------AA  102

Fzd1/2/7 reference    AACGA-GACGATCATGCCGAACTTACTCAACCACAACAGACAGGAGGAAGCCGGCTTGGA  232
Larva 2               AACGACGACGATC-------------------------------------GCAGCTTGGA  125


Fzd1/2/7 reference    CTGACACAATGGCGTTGAGAACCGGAGGAATTTTGCCGGATTTCACGGCCCACACCGTTG  60
Larva 3               CTGACACAATGGCGTTGAGAACCGGAGGAATTTTG-------------------------  35

Fzd1/2/7 reference    CGATCGTCGTCGTTTTCCTGTGCACACTCTCTGCGGTCGAATGTCAAATCGGCAGCATAT  120
Larva 3               ----CGTCGTCGTTTTCCTGTGCACACTCTCTGCGG------------TCGGCAGCATAT  79

Fzd1/2/7 reference    CGTACGGTCGTTGCGAGCCCATAACCATTCCGCTGTGCCAAGATGTCCCTTACAACGAGA  180
Larva 3               CGTACGGTC---------------------------------------------------  88

Fzd1/2/7 reference    CGATCATGCCGAACTTACTCAACCACAACAGACAGGAGGAAGCCGGCTTGGAGGTCCACC  240
Larva 3               ------------------------------------------------GGAGGTCCACC  99
```

gRNA 27 / gRNA 81 / gRNA 200

Supplementary Fig.13: Genotype of a single Fz1/2/7 mutant embryo aligned to a representative control embryo genotype sequence. All three gRNA sites are shown in three corresponding colors, with specific deletions highlighted.

FGFR

```
FgfR reference    AATCAATAAGGATGGCCGTATGATCACAGAACTGAAGAAAGTGGAAACAGAAAATGCCAA   60
Larva 1           AANCAATAAGGATGGCCGTATGATCACAGAACTGA-------------------------   35

FgfR reference    GTTTCGGTGCTCTGTTGACGCTTACCCTAAACCTAACATTACCTGGTGGAGAGGTAACGT   120
Larva 1           ------------------------------------------------------------   36

FgfR reference    CCGTCTTGAGACGGGGGATGACAGAGAGGTCAAACGGGCCACTATGACCATCAACGACGT   180
Larva 1           -------------------------AGAGGTCAAACGGGCCACTATGACCATCAACGACGT   71

FgfR reference    CGTGGTAGAAGACAGCGGTGTCTACAGCTGCTATGTAACCAATGA   225
Larva 1           CGTGGTAGA----AGCGGTGTCTACAGCTGCTANGTAACCAATGA   112


FgfR reference    AATCAATAAGGATGGCCGTATGATCACAGAACTGAAGA-AAGTGGAAACAGAAAATGCCA   59
Larva 3           AACCAANAAGGANGACNGCATGATCAC--AAC---ANAGAAGNGGAAACAGAAAATGCCA   55

FgfR reference    AGTTTCGGTGCTCTGTTGACGCTTACCCTAAACCTAACATTACCTGGTGGAGAGGTAACG   119
Larva 3           AGTTTCGGTGCTCTGTTGACGCTTACCCTAAACCTAACATTACCTGGTGGAGAGGTAACG   115

FgfR reference    TCCGTCTTGAGACGGGGGA--TGACAGAGAGGTCAAACGGGCCACTATGACCATCAACGA   177
Larva 3           TCCGTCTTGAGACGGGGGACGGGA-AGAGAGGTCAAACCGGCCAGTATGTCCATCAAGGA   174

FgfR reference    CGTCGT-GGTAGAAGACAGCGGTGTCTACAGCTGCTATGTAACCAATGA   225
Larva 3           CGT-GTAGGTAGA--------GTGTCTACAGCTGCTATGTAACCAATGA   214


FgfR reference    CAATAAGGATGGCCGTATGATCACAGAACTGAAGAAAGTGGAAACAGAAAATGCCAAGTT   60
Larva 4           CAATAAGGATGGCCGTATGATC--------------------ACAGAAAATGCCAAGTT   39

FgfR reference    TCGGTGCTCTGTTGACGCTTACCCTAAACCTAACATTACCTGGTGGAGAGGTAACGTCCG   120
Larva 4           TCGGTGCTCTGTTGACGCTTACCCTAAACCTAACATTACCTGGTGGAGAGGTAACGTCCG   99

FgfR reference    TCTTGAGACGGGGGATGACAG------------AGAGGTCAAACGGGCCACTATGACCAT   168
Larva 4           TCTTGAGACAGGGGATGATAGGGGACAGGGGATAGAGGTCAAACGGGCCACTATGACCAT   159

FgfR reference    CAACGACGTCGTGGTAGAAGACAGCGGTGTCTACAGCTGCTATGTAACCAATGA   222
Larva 4           CAACGACGTCGT-------------GGTGTCTACAGCTGCTATGTAACCAATGA   200
```

gRNA 31 / gRNA 136 / gRNA 185

Supplementary Fig.14: Genotype of a single FGFR mutant embryo aligned to a representative control embryo genotype sequence. All three gRNA sites are shown in three corresponding colors, with specific deletions highlighted.

Six1/2

```
Six1/2 reference  CTGCGAAGTTTTGCAGCAGTCGGGCAACATCGAACGCCTGGGCCGTTTTCTCTGGTCGCT  60
Larva 1           CTGCGAAGTTTTGCAGCACTCTGGCAACATCGAACGCCGGGGCCGGT-----TCGTNACC  55

Six1/2 reference  GCC---GGC--CTGCGAACACCTGCATAAGAACGAGAGT-GTGCTC----AAAG------  104
Larva 1           GCCTATTGCTTCTAGGAACAACT----------GAGGGTAGTCCTCCCGGAATGGTTCNN  105

Six1/2 reference  CCAAGGCCATTGTCGCCTTCCATCGCGGCAACTTTA------GGGAACTCTACAAGCTCT  158
Larva 1           CCTCGGCCA-TGACACCATCTATTGCTGCTACTTGAAGTCNCGGAAAGTC------CTCC  158

Six1/2 reference  TGGAAAGCAATAATTTTTCGCCA-CACAACCACCCGAAACTGCAAGC-GCTGTGGCTCAA  216
Larva 1           TGGAATG--GTAANTCTNTACCACCACCACCCCCCTAAACTGC-AGCTGCTGTGGTNCGA  215

Six1/2 reference  GGCGCACTACATCGAGGCTGAGAAACTCCGGGGCCGGCCGCTCGGAGCCGTCGGTAAATA  276
Larva 1           GGGGCACTACATCGAGGCTGAAAAACTCCAGG-----CGGTCGGATCCGTCNGTAACTA  269
```

```
Six1/2 reference  CTGCGAAGTTTTGCAGCAGTCGGGCAACATCGAACGCCTGGGCCGTTTTCTCTGGTCGCT  60
Larva 2           CTGCGAAGTTTTGCAGCAGTCGGGCAACATCGAACGCCTG----------------CGTN  44

Six1/2 reference  GCCGGCCTGCGAACACCTGCATAAGAACGAGAGTGTGCTCAAAGCCAAGGCCATTGTCGC  120
Larva 2           GCCGGCC-----------------------NAGTGTGC-NNNAGCCAAAGCCNTTGNCGC  80

Six1/2 reference  CTTCCATCGCGGCAACTTTAGGGAACTCT--------------ACAAGCTCTTGG-----  161
Larva 2           CTTCCATCGCGGCAACTTTAGGGAACTCTGGAAAACTTAAAAGAGAANTTATTGGGAGTC  140

Six1/2 reference  --AAAGCAAT---------------AATTTTTCGCCACACAACCACCCGAAACTGCAAGC  204
Larva 2           CTCAAGGAATGGTAANNCACAANCAAATGGTAC-NCACACAACCA-----------GTGC  188

Six1/2 reference  GCTGTGGCTCAAGGCGCACTACATCGAGGCTGAGAAACTC-CGGGGCCGGCCGCTCGGAG  263
Larva 2           GCTGTGGCTCTCTGCGCACTCTATCTAGGCGGACAGACTCTC---GCCGCCC-------G  238
```

```
Six1/2 reference  CTGCGAAGTTTTGCAGCAGTCGGGCAACATCGAACGCCTGGGCCGTTTTCTCTGGTCGC-  59
Larva 3           CTGCGAAGTTTTGCAGCAGTCGGGCAACATCGAACGCCTGGGCCGTT-----TGG--GCA  53

Six1/2 reference  TGCCG------GCCTGCGAACACCTGCA----TAAGAACGAGAGTGTGCTCAAAGCCAAG  109
Larva 3           GGCCGACAGTTGCCT-CG--GACCCGCAGCGTTAGGACCGGGGGG-------ACCCA--  101

Six1/2 reference  GCCATTGTCGCCT--TCCATCGCGGCAACT-TTAGGGAACTCTACAAGCTCTTGGAA---  163
Larva 3           ------CTCGCTTGGTGCTTGGC--CAAATCTTNGGG-AGTC------CTCTTGAAATGG  146

Six1/2 reference  -----------------AGCAATAATTTTTCGCCACACAACCACCCGAAACTGCAA-GCG  205
Larva 3           GACGNNCCCNNNGGGNGNGCAGTTATTTTT---CNAANNACCACCC--CACT--AAGGCG  199

Six1/2 reference  CTG-------TGGCTCAAGGCGCACTACAT-CGAGGCTGAGAAACTCCGGGGCCGGCCGC  257
Larva 3           CCGGGGGCGCGGGCCGAGGGCTAAC-AAATCCAAGGC-GAAAAACCCCCGCCCCCACCTC  257

Six1/2 reference  TCGGAGCCGTCGGTAAATACA  278
Larva 3           ACGTA-----AGGAAAAACCA  273
```

gRNA 44 / gRNA 86 / gRNA 274

Supplementary Fig.15: Genotype of a single Six1/2 mutant embryo aligned to a representative control embryo genotype sequence. All three gRNA sites are shown in three corresponding colors, with specific deletions highlighted.
