# Supplementary material for: Molecular mechanisms of tubulogenesis revealed in the sea star hydro-vascular organ
Source: Nat Commun. 2023 May 9;14:2402. doi: 10.1038/s41467-023-37947-2 (PMC10170166; doi:10.1038/s41467-023-37947-2)
Supplement: Supplementary file 2 — Description of Additional Supplementary Files [file 41467_2023_37947_MOESM2_ESM.pdf]

Description of Additional Supplementary Files

File Name: Supplementary Data 1
Description: Sequences of sgRNAs used in this study.

File Name: Supplementary Data 2
Description: amplification primers used to identify genomic mutations in CRISPR Cas9 KO larvae.

File Name: Supplementary Data 3
Description: primers used to make riboprobes from sea star larva cDNA.

File Name: Supplementary Data 4
Description: results of the RNA-seq analysis (larvae treated with FGFR inhibitor SU5402 versus controls).

File Name: Supplementary Movie 1
Description: Live imaging of tubulogenesis in the sea star embryo over the first 15 hours of tubular morphogenesis. Time lapse of a live embryo developing from gastrula to early larva. Asterisk indicates the lumen of the newly formed tubes. Time is displayed in hours:minutes:seconds; video acquired with bright field microscopy.

File Name: Supplementary Movie 2
Description: 3D movie of a sea star late larva. 3D reconstruction of a late larva from the ectoderm towards the internal organs. The main structures are annotated. Dashed line arrow indicates where the tubes meet at the anterior side to create a close system. A= anterior, P= posterior. Image taken with a 20x objective.

File Name: Supplementary Movie 3
Description: Live imaging of cell migration from the anterior side of tubes in a late gastrula. Time in hours:minutes.

File Name: Supplementary Movie 4
Description: DIC live imaging of cilia beating inside the larva left tube. Time in min:sec.

File Name: Supplementary Movie 5
Description: Movie 1; cell tracking of nuclei of stalk cells. For all Supplementary Movie s time is represented as hours:minutes and nuclei are marked by the probe H2B-GFP. Movies are maximum projections.

File Name: Supplementary Movie 6
Description: Movie 1; cell tracking of nuclei of left tube cells.

File Name: Supplementary Movie 7
Description: Movie 1; cell tracking of nuclei of right tube cells.

File Name: Supplementary Movie 8
Description: Movie 2; cell tracking of nuclei of tube cells (stalk, left and right).

File Name: Supplementary Movie 9
Description: Filopodia (magenta) extending from the tube cells during tube elongation. Time in hours:minutes.

File Name: Supplementary Movie 10
Description: Example cell tracking of a dividing cell in the growing tube. Nuclei (H2B) in cyan and actin (LifeAct) in magenta. Time in hours:minutes.

File Name: Supplementary Movie 11
Description: Contraction of the tubes in a late larva. Bright field movie of a live sea star late larva (20x objective) showing that the tube organ is vital and contracts thanks to the tube longitudinal muscles. Dashed line arrows indicate where muscle twitch happens. Particles in green are algae in the stomach. Time is indicated in seconds.
