# Supplementary material for: Molecular mechanisms of tubulogenesis revealed in the sea star hydro-vascular organ
Source: Nat Commun. 2023 May 9;14:2402. doi: 10.1038/s41467-023-37947-2 (PMC10170166; doi:10.1038/s41467-023-37947-2)
Supplement: Supplementary file 18 — Reporting Summary [file 41467_2023_37947_MOESM18_ESM.pdf]

nature portfolio

Corresponding author(s): Margherita Perillo
Gary M. Wessel

Last updated by author(s): 3-16-2023

Reporting Summary

Nature Portfolio wishes to improve the reproducibility of the work that we publish. This form provides structure for consistency and transparency in reporting. For further information on Nature Portfolio policies, see our Editorial Policies and the Editorial Policy Checklist.

Statistics

For all statistical analyses, confirm that the following items are present in the figure legend, table legend, main text, or Methods section.

n/a	Confirmed	
☐	☒	The exact sample size (*n*) for each experimental group/condition, given as a discrete number and unit of measurement
☐	☒	A statement on whether measurements were taken from distinct samples or whether the same sample was measured repeatedly
☐	☒	The statistical test(s) used AND whether they are one- or two-sided<br>*Only common tests should be described solely by name; describe more complex techniques in the Methods section.*
☐	☒	A description of all covariates tested
☐	☒	A description of any assumptions or corrections, such as tests of normality and adjustment for multiple comparisons
☐	☒	A full description of the statistical parameters including central tendency (e.g. means) or other basic estimates (e.g. regression coefficient) AND variation (e.g. standard deviation) or associated estimates of uncertainty (e.g. confidence intervals)
☐	☒	For null hypothesis testing, the test statistic (e.g. *F*, *t*, *r*) with confidence intervals, effect sizes, degrees of freedom and *P* value noted<br>*Give P values as exact values whenever suitable.*
☒	☐	For Bayesian analysis, information on the choice of priors and Markov chain Monte Carlo settings
☒	☐	For hierarchical and complex designs, identification of the appropriate level for tests and full reporting of outcomes
☒	☐	Estimates of effect sizes (e.g. Cohen's *d*, Pearson's *r*), indicating how they were calculated

Our web collection on statistics for biologists contains articles on many of the points above.

Software and code

Policy information about availability of computer code

Data collection	Microscope image data was collected from the associated software linked to each microscope: Olympus Fluoview (V4.2), Zeiss Zen (Zen 2.5 Blue Edition version 2.5.75.0) and Nikon NIS Elements (Advanced Research Package, NIS Elements version 5.21.03). For qPCR: QuantStudio3 (ThermoFisher, V2.6.0).
Data analysis	Graph Pad Prism (San Diego, CA)(V. 7.0.0); FIJI (https://fiji.sc/) (ImageJ 1.53c); Excel (Office 365 V.16.70); TIDE (Tracking of Indels by Decomposition https://academic.oup.com/nar/article/42/22/e168/2411890, V. 3.3.0); SynthegoICE (V.3, https://ice.synthego.com/#); GeoRose (https://www.yongtechnology.com) (V. 0.5.1.1); Adobe Illustrator (V. 27.2); Interproscan V. 5.38-76.0.

For manuscripts utilizing custom algorithms or software that are central to the research but not yet described in published literature, software must be made available to editors and reviewers. We strongly encourage code deposition in a community repository (e.g. GitHub). See the Nature Portfolio guidelines for submitting code & software for further information.

Data

Policy information about availability of data

All manuscripts must include a data availability statement. This statement should provide the following information, where applicable:

- Accession codes, unique identifiers, or web links for publicly available datasets
- A description of any restrictions on data availability
- For clinical datasets or third party data, please ensure that the statement adheres to our policy

The RNA-seq data generated in this study have been deposited in the NCBI BioProject database under accession code PRJNA898435 [https://www.ncbi.nlm.nih.gov/bioproject/?term=PRJNA898435]. The processed RNA-seq data and the GOterm analysis results are provided as Supplementary Data 4. The Supplementary Movie s in Figure 2 and 3 analyzed in this study are provided as Supplementary Movie 5-8. The Supplementary Movie used for images in Figure 1g are provided in Supplementary Movie 4; the Supplementary Movie used for Figure 2i is provided in Supplementary Movie 10; Supplementary Movies used to make Figure 3g-h are provided in Supplementary Movie 9. Source data are provided with this paper.

Human research participants

Policy information about studies involving human research participants and Sex and Gender in Research.

Reporting on sex and gender	N/A
Population characteristics	N/A
Recruitment	N/A
Ethics oversight	N/A

Note that full information on the approval of the study protocol must also be provided in the manuscript.

Field-specific reporting

Please select the one below that is the best fit for your research. If you are not sure, read the appropriate sections before making your selection.

☒ Life sciences ☐ Behavioural & social sciences ☐ Ecological, evolutionary & environmental sciences

For a reference copy of the document with all sections, see nature.com/documents/nr-reporting-summary-flat.pdf

Life sciences study design

All studies must disclose on these points even when the disclosure is negative.

Sample size	For every experiment multiple factors were analyzed to test the significance of the results. Sample size was chosen based on the number of embryos sufficient to perform statistical student t-test. In our field, around 15-50 sea star embryos are used for each experiment. Similar datasets were used in "Minyan Zheng Olga Zueva Veronica F Hinman (2022) Regeneration of the larval sea star nervous system by wounding induced respecification to the Sox2 lineage eLife 11:e72983.https://doi.org/10.7554/eLife.72983"
Data exclusions	No data were excluded.
Replication	Each experiment was repeated between 3 and 5 times, for some experiemnts even more. All attempts at replication were successful.
Randomization	All sea star embryos used for these experiments derive from at least three different females and males, therefore in every experiment there is a mix of genotypes. Adult sea stars do not show any phenotypic trait that can differentiate among individuals, therefore animals were randomly chosen. The sex of the larvae is always unknown and individual larvae with the same genotype were identical, therefore allocation in experimental groups was not relevant in our study.
Blinding	Embryo handling and advanced live imaging requires a highly trained skill and only one investigator was qualified to generate the mutants and analyse the phenotype. However, the genotyping and the analysis of the results were performed by the other authors. Furthermore, to control for bias we performed precise measurements and statistical tests, as it is standard practice in our field.

Reporting for specific materials, systems and methods

We require information from authors about some types of materials, experimental systems and methods used in many studies. Here, indicate whether each material, system or method listed is relevant to your study. If you are not sure if a list item applies to your research, read the appropriate section before selecting a response.

Materials & experimental systems

n/a	Involved in the study
☐	☒ Antibodies
☒	☐ Eukaryotic cell lines
☒	☐ Palaeontology and archaeology
☐	☒ Animals and other organisms
☒	☐ Clinical data
☒	☐ Dual use research of concern

Methods

n/a	Involved in the study
☒	☐ ChIP-seq
☒	☐ Flow cytometry
☒	☐ MRI-based neuroimaging

Antibodies

Antibodies used

Alexa-fluor 488 Phalloidin 1:300 (Molecular Probes, Cat.#A12379), anti-beta tubulin antibody 1:100 (Hybridoma bank, Cat.#E7), anti-laminin antibody 1:300 (Abcam, Cat.#AB11575), monoclonal Anti-MAP Kinase, Activated (Diphosphorylated ERK-1&2) pERK 1:100 (Millipore Sigma, Cat.#M8159). AlexaFluo secondary antibodies (Invitrogen anti-mouse Cat.#A11001 and anti-rabbit Cat.#A-11037 1:2000.

Validation

Primary antibodies in this paper have been validated and are widely used across organisms and applications. Validations were performed by the manufacturer: anti-beta tubulin antibody(https://dshb.biology.uiowa.edu/E7_2; E7,130 citations); anti laminin: https://www.abcam.com/laminin-antibody-ab11575.html; ab11575, 458 citations); anti pERK: https://www .sigmaal drich .com/US/en/product/sigma/m8159, M8159, 345 citations)

Animals and other research organisms

Policy information about studies involving animals; ARRIVE guidelines recommended for reporting animal research, and Sex and Gender in Research

Laboratory animals

Sea stars Patiria miniata are collected by divers in the Pacific Coast off of Southern Californa. No sex or age information is available.

Wild animals

No wild animals were used in the study.

Reporting on sex

No sex or age information is available for sea star embryos and larvae.

Field-collected samples

Sea stars Patiria miniata are collected by divers in the Pacific Coast off of Southern Californa. No sex or age information is available.No field collected samples were used in the study.

Ethics oversight

No ethical approval was required in the study.

Note that full information on the approval of the study protocol must also be provided in the manuscript.
